# Morphological and multilocus phylogenetic analysis of *Sirococcus* (*Diaporthales*, *Gnomoniaceae*) on woody hosts from Fujian, Shandong, and Yunnan, China: Two new species and two new records

**DOI:** 10.3897/mycokeys.142.207158

**Published:** 2026-10-27

**Authors:** Yaling Wang, Qiyun Liu, Mengyuan Zhang, Zhaoxue Zhang, Xiuguo Zhang, Zhuang Li

**Affiliations:** 1 College of Plant Protection, Shandong Agricultural University, Taian, Shandong, 271018, China College of Plant Protection, Shandong Agricultural University Taian China

**Keywords:** Fungal biodiversity, fungal taxonomy, plant-associated fungi

## Abstract

*Sirococcus* (*Diaporthales*, *Gnomoniaceae*) has recently been recircumscribed based on multilocus phylogenetic and morphological evidence, with all species formerly placed in *Gnomoniopsis* transferred to *Sirococcus* under the principle of nomenclatural priority. In this study, we investigated the species diversity of *Sirococcus* associated with native woody hosts from Fujian, Shandong, and Yunnan provinces, China. A total of six isolates were recovered from leaf samples of plants belonging to *Fagaceae*, *Lauraceae*, and *Theaceae*. Phylogenetic analysis based on concatenated ITS, *tef1*, and *tub2* sequence datasets resolved these isolates into four *Sirococcus* species: two new species (*S. euryicola***sp. nov**. and *S. linderae***sp. nov**.), one new host record for *S. fujianensis*, and one new geographical record for *S. mengyinensis*. We provide detailed morphological descriptions, photographic illustrations, and multilocus sequence data for all four species, along with compiled host and distribution notes, and discuss their phylogenetic affinities and taxonomic implications within the genus. Our findings substantially expand the known species diversity, host range, and geographical distribution of *Sirococcus* in China, and highlight the underexplored diversity of this genus on non-*Fagaceae* woody hosts.

## Introduction

*Diaporthales* (*Ascomycota*, *Sordariomycetes*) is a species-rich fungal order with diverse ecological roles and substantial economic relevance (Senanayake et al. 2017, 2018; Fan et al. 2018; Jiang et al. 2019a, 2024, 2025a). Members of *Diaporthales* occur as plant pathogens, endophytes, or saprobes decomposing leaf litter and woody debris, colonising a wide array of woody and herbaceous hosts (Jiang et al. 2021a, 2025b; Zhu et al. 2022; Du et al. 2025). Within the order, generic delimitation in the family *Gnomoniaceae* has long been taxonomically challenging, and the generic boundary between *Sirococcus* and *Gnomoniopsis* has been particularly disputed historically, until a recent comprehensive taxonomic revision resolved this conflict (Jiang et al. 2026).

*Sirococcus* was established by Preuss in 1855 with *S. conigenus* as the type species (Preuss 1855; Sutton 1980; Cannon and Minter 1983). Historically, *Sirococcus* were predominantly known from asexual morphs infecting coniferous trees (Preuss 1855; Bahnweg et al. 2000; Rossman et al. 2008), whereas *Gnomoniopsis*, established by Berlese in 1893, were characterised by sexual morphs on broadleaf hosts (Berlese 1893; Sogonov et al. 2008; Walker et al. 2010; Jiang and Tian 2019). For over a century, the two genera were treated as separate taxa based on the predominant expression of their asexual and sexual morphs, respectively.

However, accumulating molecular phylogenetic evidence has challenged this traditional morphology-based classification. Multiple independent studies confirmed that species of the two genera form a single, well-supported monophyletic clade within *Gnomoniaceae* (Rossman et al. 2008; Senanayake et al. 2018; Jiang et al. 2019b; Yang et al. 2020). Most recently, Jiang et al. (2026) conducted the most comprehensive re-evaluation of the generic boundaries between *Sirococcus* and *Gnomoniopsis*, using a concatenated three-locus dataset (ITS, *tef1*, and *tub2*). Their phylogenetic analysis revealed that all species of *Gnomoniopsis* nested within *Sirococcus*, forming a single, robustly supported monophyletic lineage. Furthermore, they demonstrated that the asexual morphological characteristics of species from both genera were indistinguishable. Based on these findings and the principle of priority under the International Code of Nomenclature for algae, fungi, and plants (ICN; Hawksworth et al. 2009; Rossman 2014), Jiang et al. (2026) formally synonymised *Gnomoniopsis* under *Sirococcus* and proposed 38 new combinations.

*Sirococcus* are widely distributed in temperate and subtropical regions and colonise a broad range of host plants. They exhibit diverse trophic lifestyles, functioning as saprobes, endophytes, or plant pathogens, and are responsible for various disease symptoms, including shoot blight, leaf spots, and fruit rot (Bahnweg et al. 2000; Rossman et al. 2008; Shuttleworth and Guest 2017; Çakar 2024; Jiang and Tian 2019). Several species are of considerable economic importance as plant pathogens. For example, *Sirococcus daii* (basionym *Gnomoniopsis daii*) causes severe nut rot of *Castanea mollissima* in China (Jiang and Tian 2019). *Sirococcus smithogilvyi* (basionym *Gnomoniopsis smithogilvyi*; syn. *G. castaneae*) is the primary causal agent of sweet chestnut brown rot (Visentin et al. 2012; Shuttleworth et al. 2016; Lema et al. 2023). This pathogen causes severe nut rot and cankers on *Castanea*, threatening chestnut production in Europe, North America, and Oceania (Shuttleworth and Guest 2017; Çakar 2024).

Despite the recent major taxonomic revision of the genus, the species diversity of *Sirococcus* in China remains insufficiently explored. Most previous studies have focused predominantly on chestnut-associated pathogenic species, whereas *Sirococcus* taxa associated with other hosts are poorly documented. Accordingly, the present study aimed to: (1) investigate the species diversity of *Sirococcus* associated with diverse hosts plants across three provinces of China (Fujian, Shandong, and Yunnan); (2) characterise and describe novel species based on morphological observations and multilocus phylogenetic analysis; (3) evaluate potential conspecificity of closely related taxa *S. xishuangbannaensis*–*S. yunnanensis* and *S. taishanensis*–*S. chinensis*; and (4) supplement morphological descriptions and photographic illustrations for newly recorded hosts and provide geographical distributions of known *Sirococcus* species.

## Materials and methods

### Strain isolation and preservation

Plant specimens with symptomatic and necrotic leaves were sampled from three provinces in China (Fujian, Shandong, and Yunnan) between 2024 and 2025. All collections were labelled with the collection date, locality, and host identity, and were photographed and processed within 24 h. Pure cultures were isolated using the tissue segment method (Zhang et al. 2024; Li et al. 2025). Briefly, 5 × 5 mm tissue pieces were cut from the lesion margins of 5–7 leaves per sample. Surface sterilisation was conducted as follows: 1 min in 75% ethanol, 30 s in sterile water, 1 min in 5% sodium hypochlorite, followed by three final rinses in sterile water (30 s each). Sterilised tissue segments were blotted dry on sterile filter paper and plated on potato dextrose agar (PDA). Plates were incubated at 25 °C in the dark for 3 d. Pure cultures were obtained by transferring hyphal tips from colony edges to fresh PDA plates. All isolates were maintained in 15% (v/v) sterile glycerol at 4 °C for subsequent analyses. Holotype and paratype specimens are deposited in HSAUP (Herbarium of Shandong Agricultural University, Tai’an). Ex-type cultures have been deposited in SAUCC (Shandong Agricultural University Culture Collection) and CGMCC (China General Microbiological Culture Collection Center).

### Morphological characterisation

All isolates were grown on potato dextrose agar (PDA). PDA was prepared by boiling 200 g peeled potatoes in 1 L of distilled water for 30 min, adding 20 g dextrose and 20 g agar, and adjusting to pH 7.0. Colony characteristics, pigmentation, and growth rates were documented after 7 d of incubation. Colony obverse and reverse views were photographed with a Canon PowerShot G7X (Canon Inc., Tokyo, Japan). Sporulation was induced on sterile pine needles placed on water agar (WA). Conidial morphology, dimensions, and septation were examined. Macrostructures were observed under an Olympus SZ61 stereomicroscope, and microstructures under an Olympus BX53 compound microscope with differential interference contrast (DIC). Images were captured using mounted BioHD-A20c digital cameras (FluoCa Scientific, Shanghai, China). Microstructures were measured from randomly selected mature conidia using Digimizer v5.6.0 (https://www.digimizer.com, accessed on 10 September 2026); mean values ± standard deviation were calculated for each taxon.

### DNA extraction, amplification, and sequencing

Genomic DNA was extracted from 7-d-old PDA-grown mycelia using the CTAB method (Wang et al. 2023). Three loci were amplified by PCR: ITS, partial *tef1*, and partial *tub2*, using primer pairs ITS5/ITS4, EF1-728F/EF-2, and Bt-2a/Bt-2b, respectively (Table 1). PCR amplifications were carried out in 25 μL reactions containing 12.5 μL 2× Hieff Canace Plus Master Mix (Yeasen, Shanghai, China), 1 μL of each primer (10 μM; TsingKe, Qingdao, China), 1 μL template DNA (~50 ng μL^-1^), and ddH_2_O to a final volume of 25 μL. Amplicons were separated on 1% agarose gels stained with Safe Red (ABclonal, Wuhan, China) and visualised under UV light. Target DNA bands were excised from the gels and purified with a gel extraction kit (DM1200, Beijing Solarbio Science & Technology Co., Ltd.), followed by bidirectional Sanger sequencing at Youkang Biotechnology (Zhejiang, China). Consensus sequences were assembled from forward and reverse chromatograms. Preliminary searches using BLASTn (https://blast.ncbi.nlm.nih.gov/Blast.cgi) against the NCBI GenBank nucleotide database were performed for each newly generated sequence to identify closely related taxa and assist the selection of reference sequences for phylogenetic analysis. Sequence alignments were performed in MEGA v7.0 (Kumar et al. 2016). All sequences generated in this study have been deposited in GenBank under the accession numbers provided in Table 2.

**Table 1.** Gene loci and corresponding PCR primers and programs used in this study.

**Loci**	**PCR primers**	**Sequences (5**'→**3**')	**PCR cycles**	**References**
ITS	ITS5	GGA AGT AAA AGT CGT AAC AAG G	95 °C: 5 min; (95 °C: 30s, 55 °C: 30s, 72 °C: 1 min) × 35 cycles	White et al. 1990
	ITS4	TCC TCC GCT TAT TGA TAT GC		
*tef1*	EF1-728F	CAT CGA GAA GTT CGA GAA GG	95 °C: 5 min; (95 °C: 30s, 48 °C or 55 °C: 60s, 72 °C: 1 min) × 30 cycles	O’Donnell et al. 1998; Carbone and Kohn 1999
	EF2	GGA RGT ACC AGT SAT CAT GTT		
*tub2*	Bt-2a	GGT AAC CAA ATC GGT GCT GCT TTC	95 °C: 5 min; (95 °C: 30s, 53 °C: 30s, 72 °C: 1 min) × 35 cycles	Glass and Donaldson 1995
	Bt-2b	ACC CTC AGT GTA GTG ACC CTT GGC		

**Table 2.** GenBank accession numbers of the taxa in the phylogenetic analysis.

**Species**	**Country**	**Host**	**Host Family**	**Strain**	**GenBank Accession Number**			**References**
					**ITS**	***tef1***	***tub2***	
*Apiognomonia errabunda*	Switzerland	*Fagus sylvatica*	* Fagaceae *	AR 2813	DQ313525	DQ313565	DQ862014	Sogonov et al. 2007
*Discula destructiva*	USA	*Cornus nuttallii*	* Cornaceae *	CBS 109771	EF512464	EF512526	NA	Rossman et al. 2008
		*Cornus florida*		AR 2817	EF512465	EF512527	NA	
*Sirococcus alderdunensis*	USA	*Rubus pedatus*	* Rosaceae *	CBS 125679	GU320826	GU320813	GU320788	Walker et al. 2010
		*Rubus parviflorus*		CBS 125680*	GU320825	GU320801	GU320787	
				CBS 125681	GU320827	GU320802	GU320789	
*S. angolensis*	Angola	Unknown	Unknown	CBS 145057*	MK047428	NA	NA	Crous et al. 2018
*S. annonae*	China	*Annona montana*	* Annonaceae *	CGMCC 3.28195*	PQ325623	PQ336654	PQ336678	Zhang et al. 2025
				SAUCC 6333	PQ325624	PQ336655	PQ336679	
				CGMCC 3.28194	PQ325625	PQ336656	PQ336680	
				SAUCC 6423	PQ325626	PQ336657	PQ336681	
*S. castaneae*	Switzerland	*Castanea sativa*	* Fagaceae *	CBS 142041	KX929744	KX929710	KX958443	Meyer et al. 2017
*S. castanopsidis*	China	*Castanopsis hystrix*	* Fagaceae *	CFCC 54437*	MZ902909	MZ936385	NA	Jiang et al. 2021b
				CFCC 55878	MZ902910	MZ936386	NA	
*S. chamaemori*	Finland	*Rubus chamaemorus*	* Rosaceae *	CBS 804.79	GU320817	GU320809	GU320777	Walker et al. 2010
*S. chinensis*	China	*Castanea mollissima*	* Fagaceae *	CFCC 52286*	MG866032	MH545370	MH545366	Jiang et al. 2020
				CFCC 52287	MG866033	MH545371	MH545367	
				CFCC 52288	MG866034	MH545372	MH545368	
				CFCC 52289	MG866035	MH545373	MH545369	
*S. clavulatus*	USA	*Quercus falcata*	* Fagaceae *	CBS 121255	EU254818	GU320807	EU219211	Sogonov et al. 2008
*S. comari*	Finland	*Comarum palustre*	* Rosaceae *	CBS 806.79	EU254821	GU320810	EU219156	Sogonov et al. 2008
				CBS 807.79	EU254822	GU320814	GU320779	
	Switzerland			CBS 809.79	EU254823	GU320794	GU320778	
*S. conigenus*	Finland	*Picea abies*	* Pinaceae *	CBS 119615*	EF512470	EF512532	NA	Rossman et al. 2008
	Canada	*Larix laricina*		DAOM 191767	EF512483	EF512545	NA	
				CBS 119602	EF512467	EF512529	NA	
	Germany	*Picea pungens*		CBS 113.75	EF512482	EF512544	EU219129	
*S. daii*	China	*Castanea mollissima*	* Fagaceae *	CFCC 54043*	MN598671	MN605519	MN605517	Jiang and Tian 2019
		*Castanopsis fargesii*		CGMCC 3.28193	PQ325627	PQ336658	PQ336682	Zhang et al. 2025
				SAUCC 0153	PQ325628	PQ336659	PQ336683	
		*Castanea mollissima*		CFCC 54345	MW208113	MW227345	MW218543	Jiang et al. 2021c
*S. diaoluoshanensis*	China	*Castanopsis chinensis*	* Fagaceae *	SAUCC DL0963*	ON753744	ON759769	ON759777	Wang et al. 2022
				SAUCC DL0964	ON753743	ON759768	ON759776	
*S. euryae*	China	*Eurya nitida*	* Theaceae *	CGMCC 3.28234*	PQ325629	PQ336660	PQ336684	Zhang et al. 2025
				SAUCC 3977	PQ325630	PQ336661	PQ336685	
*S. euryicola*	**China**	***Eurya japonica***	*** Theaceae ***	CGMCC 3.40298*	**PZ575042**	**PZ675984**	**PZ675992**	**This study**
				**SAUCC 8420**	**PZ575041**	**PZ675983**	**PZ675991**	
*S. fagacearum*	China	*Lithocarpus glaber*	* Fagaceae *	CFCC 54316*	MZ902916	MZ936392	MZ936408	Jiang et al. 2021b
*S. flavus*	China	*Castanopsis carlesii*	* Fagaceae *	CFCC 71563*	PV257808	PV268106	PV339811	Li et al. 2025
				CFCC 71566	PV257809	PV268107	PV339812	
				CFCC 71567	PV257810	PV268108	PV339813	
*S. fragariae*	USA	*Fragaria vesca*	* Rosaceae *	CBS 121226	EU254824	GU320792	EU219144	Sogonov et al. 2008
	France	*Fragaria* sp.		CBS 208.34	EU254826	GU320808	EU219149	
	USA			CBS 125671	GU320816	GU320793	GU320776	
*S. fujianensis*	**China**	*Castanopsis chinensis*	* Fagaceae *	CGMCC 3.28229*	PQ325641	PQ336672	PQ336696	Zhang et al. 2025
				SAUCC 1260	PQ325642	PQ336673	PQ336697	
		***Camellia sinensis***	*** Theaceae ***	**SAUCC 10881**	**PZ575039**	**PZ675981**	**PZ675989**	**This study**
*S. guangdongensis*	China	*Castanopsis fargesii*	* Fagaceae *	CFCC 54443*	MZ902918	MZ936394	MZ936410	Jiang et al. 2021b
				CFCC 54331	MZ902919	MZ936395	MZ936411	
				CFCC 54282	MZ902920	MZ936396	MZ936412	
*S. guttulatus*	Bulgaria	*Agrimonia eupatoria*	* Rosaceae *	MS 0312	EU254812	NA	NA	Sogonov et al. 2008
*S. hainanensis*	China	*Castanopsis hainanensis*	* Fagaceae *	CFCC 54376*	MZ902921	MZ936397	MZ936413	Jiang et al. 2021b
				CFCC 55877	MZ902922	MZ936398	MZ936414	
*S. idaeicola*	USA	*Rubus* sp.	* Rosaceae *	CBS 125672	GU320823	GU320797	GU320781	Walker et al. 2010
		*Rubus pedatus*		CBS 125673	GU320824	GU320798	GU320782	
	France	*Rubus* sp.		CBS 125674*	GU320820	GU320796	GU320780	
	USA	*Rubus pedatus*		CBS 125675	GU320822	GU320799	GU320783	
				CBS 125676	GU320821	GU320811	GU320784	
*S. juglandis*	China	*Juglans regia*	* Juglandaceae *	CGMCC 3.28233*	PQ325637	PQ336668	PQ336692	Zhang et al. 2025
				SAUCC 2375	PQ325638	PQ336669	PQ336693	
*S. lanceolatae*	China	*Phoebe lanceolata*	* Lauraceae *	CGMCC 3.28232*	PQ325639	PQ336670	PQ336694	Zhang et al. 2025
				SAUCC 1277	PQ325640	PQ336671	PQ336695	
*S. lithocarpi*	China	*Lithocarpus fohaiensis*	* Fagaceae *	SAUCC YN0743*	ON753749	ON759765	ON759783	Wang et al. 2022
				SAUCC YN0742	ON753750	ON759764	ON759782	
*S. linderae*	**China**	***Lindera aggregata***	*** Lauraceae ***	CGMCC 3.40299*	**PZ575038**	**PZ675980**	**PZ675988**	**This study**
				**SAUCC 11015**	**PZ575037**	**PZ675979**	**PZ675987**	
*S. macounii*	USA	*Spiraea* sp.	* Rosaceae *	CBS 121468	EU254762	GU320804	EU219126	Sogonov et al. 2008
*S. melastomatis*	China	*Melastoma candidum*	* Melastomataceae *	CGMCC 3.28231*	PQ325631	PQ336662	PQ336686	Zhang et al. 2025
				SAUCC 0031	PQ325632	PQ336663	PQ336687	
*S. mengyinensis*	**China**	***Castanea mollissima***	*** Fagaceae ***	SAUCC MY0293*	ON753741	ON759766	ON759774	Wang et al. 2022
				SAUCC MY0296	ON753742	ON759767	ON759775	
				CFCC 71876	PX982831	PX981897	PX981909	Jiang et al. 2026
				CFCC 71877	PX982832	PX981898	PX981910	
				CFCC 71908	PX982833	PX981899	PX981911	
				**SAUCC 10843**	**PZ575040**	**PZ675982**	**PZ675990**	**This study**
*S. occultus*	USA	*Potentilla* sp.	* Rosaceae *	CBS 125677	GU320828	GU320812	GU320785	Walker et al. 2010
				CBS 125678*	GU320829	GU320800	GU320786	
*S. paraclavulatus*	USA	*Quercus alba*	* Fagaceae *	CBS 123202	GU320830	GU320815	GU320775	Walker et al. 2010
*S. piceicola*	Canada	*Picea sitchensis*	* Pinaceae *	CBS 119620*	EF512480	EF512542	EU219130	Rossman et al. 2008
		*Picea glauca*		CBS 119625	EF512475	EF512537	NA	
	Switzerland	*Picea abies*		CBS 119621	EF512474	EF512536	NA	
*S. quercus*	USA	*Quercus* sp.	* Fagaceae *	CBS 142126	KY173465	NA	NA	Crous et al. 2013
*S. racemulus*	USA	*Chamerion angustifolium*	* Onagraceae *	CBS 121469*	EU254841	GU320803	EU219125	Sogonov et al. 2008
*S. rosae*	New Zealand	*Rosa* sp.	* Rosaceae *	CBS 145085*	MK047451	NA	NA	Crous et al. 2018
	China	*Rosa chinensis*		CFCC 72007	PX982834	PX981900	PX981912	Jiang et al. 2026
*S. rossmaniae*	China	*Castanopsis hainanensis*	* Fagaceae *	CFCC 54307*	MZ902923	MZ936399	MZ936415	Jiang et al. 2021b
				CFCC 55876	MZ902924	MZ936400	MZ936416	
*S. sanguisorbae*	Switzerland	*Sanguisorba minor*	* Rosaceae *	CBS 858.79	GU320818	GU320805	GU320790	Walker et al. 2010
*S. saprophyticus*	China	*Melastoma candidum*	* Melastomataceae *	SAUCC 3040	PQ325633	PQ336664	PQ336688	Zhang et al. 2025
				SAUCC 0051	PQ325634	PQ336665	PQ336689	
*S. silvicola*	China	*Castanopsis hystrix*	* Fagaceae *	CFCC 54304	MZ902925	MZ936401	MZ936417	Jiang et al. 2021b
		*Quercus serrata*		CFCC 54418*	MZ902926	MZ936402	MZ936418	
		*Castanopsis carlesii*		CFCC 71885	PX982835	PX981901	PX981913	Jiang et al. 2026
*S. smithogilvyi*	Australia	*Castanea* sp.	* Fagaceae *	CBS 130190*	JQ910642	KR072534	JQ910639	Crous et al. 2012
				CBS 130189	JQ910644	KR072535	JQ910641	
				CBS 130188	JQ910643	KR072536	JQ910640	
	Italy	*Castanea sativa*		MUT 401	HM142946	KR072537	KR072532	Visentin et al. 2012
	New Zealand			MUT 411	HM142948	KR072538	KR072533	
*S. taishanensis*	China	*Castanea mollissima*	* Fagaceae *	CGMCC 3.28230*	PQ325645	PQ336676	PQ336700	Zhang et al. 2025
				SAUCC 1033	PQ325646	PQ336677	PQ336701	
*S. tormentillae*	Switzerland	*Potentilla* sp.	* Rosaceae *	CBS 904.79	EU254856	GU320795	EU219165	Sogonov et al. 2008
*S. tsugae*	USA	*Tsuga heterophylla*	* Pinaceae *	CBS 119617	EF512466	EF512528	NA	Rossman et al. 2008
		*Cedrus deodara*		CBS 119618	EF512479	EF512541	NA	
	NA	NA	NA	CBS 128356	JF514853	JF514834	JF514844	Walker et al. 2012
*S. xishuangbannaensis*	China	*Castanea mollissima*	* Fagaceae *	CGMCC 3.25980*	PQ325643	PQ336674	PQ336698	Zhang et al. 2025
				CGMCC 3.25979	PQ325644	PQ336675	PQ336699	
*S. xunwuensis*	China	*Castanopsis fissa*	* Fagaceae *	CFCC 53115*	MK432667	MK578141	MK578067	Yang et al. 2020
				CFCC 53116	MK432668	MK578142	MK578068	
*S. yunnanensis*	China	*Castanea mollissima*	* Fagaceae *	SAUCC YN1659*	ON753746	ON759771	ON759779	Wang et al. 2022
				SAUCC YN1657	ON753747	ON759772	ON759780	

Notes: New species described and new records reported in this study are indicated in bold. Taxa marked with an asterisk (*) represent ex-type or ex-epitype strains. NA = not available. Taxonomic authorities for all fungal and plant species listed in this table are provided in Suppl. material 1.

### Phylogenetic analysis

Sequence alignments were performed using MAFFT v7 (available at http://mafft.cbrc.jp/alignment/server/) and manually refined in MEGA v7.0 (Katoh et al. 2019). Phylogenetic analysis was conducted using maximum-likelihood (ML) and Bayesian inference (BI) based on the concatenated alignment of the ITS, *tef1*, and *tub2* gene regions. Maximum-likelihood analysis was implemented in RAxML-NG v1.1.0 under the GTR+G+I nucleotide substitution model. The final alignment comprised 110 taxa with a total of 2,474 nucleotide characters (including gaps). Tree searches were carried out with 20 independent starting trees (10 random trees and 10 maximum-parsimony trees) to ensure recovery of the globally optimal topology.

Bayesian phylogenetic inference was performed using MrBayes v3.2.7 (Ronquist et al. 2012). Partition-specific nucleotide substitution models were applied to the three gene partitions: GTR+I+G for ITS and *tef1*, and GTR+G for *tub2*. Rate multipliers were used to account for relative evolutionary rate differences across partitions. Two independent Markov chain Monte Carlo (MCMC) runs were set up, each containing one cold chain and three heated chains with a temperature parameter of 0.10. Runs were executed for a total of 5,000,000 generations, with sampling of trees and model parameters every 1,000 generations. After discarding the first 25% of samples as burn-in, a 50% majority-rule consensus tree was constructed from the remaining 7,502 sampled trees to calculate clade posterior probabilities (PP). Convergence was assessed comprehensively based on the average standard deviation of split frequencies (ASDSF), potential scale reduction factor (PSRF), and effective sample size (ESS) of all parameters. Runs were considered converged when the ASDSF was < 0.01, ESS values for all parameters were > 100, and PSRF values approached 1.0. Phylogenetic trees were visualised in FigTree v1.4.2 (Rambaut 2014) and further edited for publication using Adobe Illustrator.

## Results

### Phylogenetic analysis

**Figure 1.**
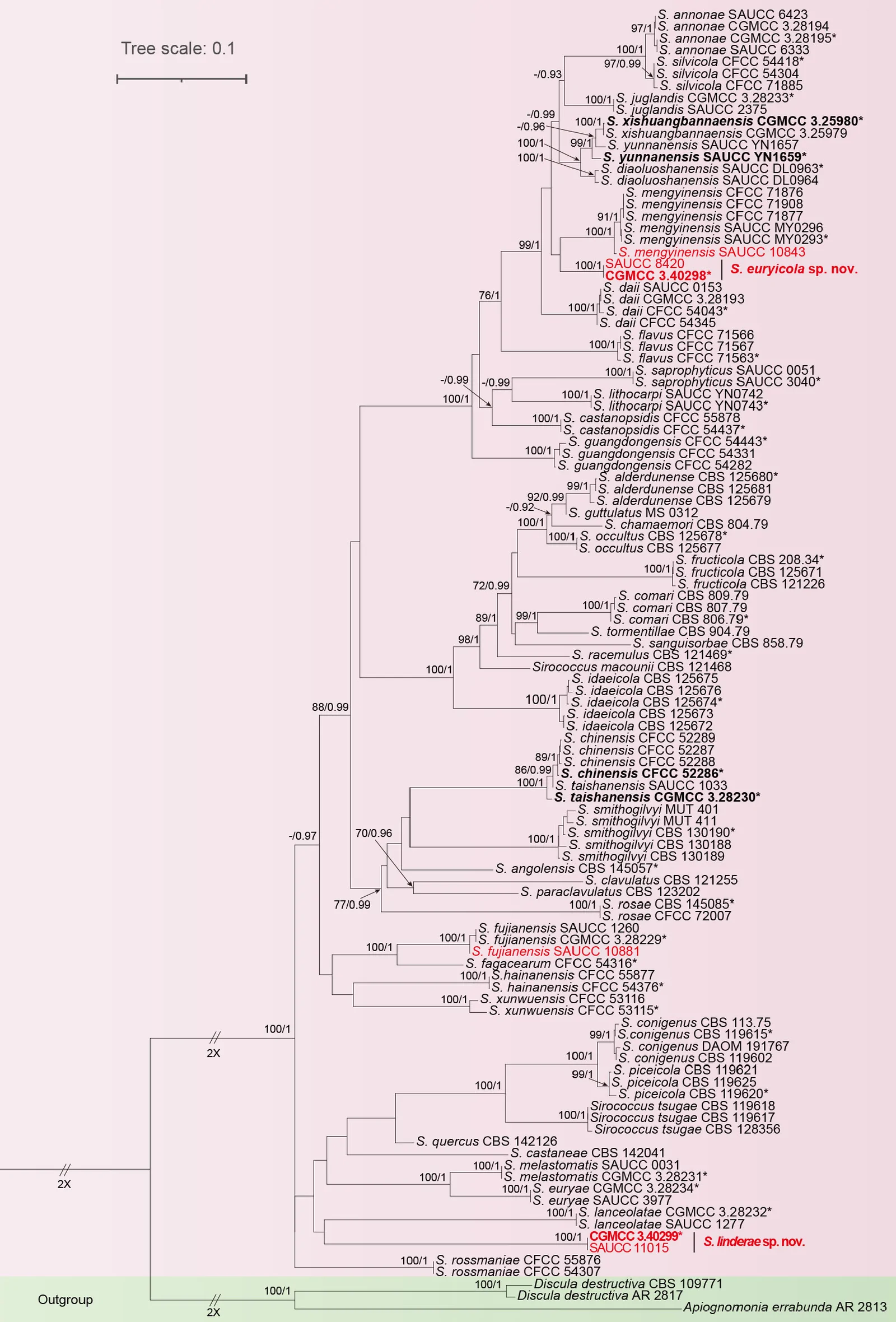
Maximum-likelihood tree of *Sirococcus* species inferred from the combined ITS, *tef1*, and *tub2* sequence dataset. Nodal support values are shown as ML bootstrap support (≥ 70%) followed by Bayesian posterior probability (≥ 0.90). *Apiognomonia errabunda* (AR 2813) and *Discula destructiva* (CBS 109771, AR 2817) were used as outgroups. Asterisks (*) represent ex-type or ex-epitype strains. Strains obtained in this study are highlighted in red. The ex-type strains of the four species further evaluated in the Critical evaluation section (*S. xishuangbannaensis*, *S. yunnanensis*, *S. taishanensis*, and *S. chinensis*) are shown in bold black.

The phylogenetic dataset was constructed based on the combined sequences of three nuclear gene fragments: ITS, *tef1*, and *tub2*, comprising a total of 110 sequences. After sequence alignment and trimming of poorly aligned regions, the final effective alignment length was 2,474 bp (including gaps), partitioned by gene as follows: ITS (positions 1–649), *tef1* (positions 650–1,425), and *tub2* (positions 1,426–2,474). Gaps accounted for 38.77% of the total alignment length, while conserved invariable sites accounted for 47.16%. Phylogenetic trees were inferred using maximum likelihood (ML) and Bayesian inference (BI) methods. The topologies yielded by the two algorithms were highly consistent overall, with only minor discrepancies in the arrangement of some low-support terminal branches. The ML tree is presented as the primary result in this study, with ML bootstrap support (BS) and Bayesian posterior probability (PP) values labelled at nodes; only BS values ≥ 70% and PP values ≥ 0.90 are shown (Fig. 1).

Phylogenetic topology revealed that the genus *Sirococcus* formed a well-supported monophyletic clade in both analyses (MLBS = 100%, BIPP = 1.00), clearly separated from the outgroups *Apiognomonia errabunda* (AR 2813) and *Discula destructiva* (CBS 109771, AR 2817). Within *Sirococcus*, the six strains obtained in this study were resolved into four species. *Sirococcus euryicola* (CGMCC 3.40298) formed a distinct, highly supported monophyletic clade (MLBS = 100%, BIPP = 1.00) and was sister to *S. mengyinensis*. *Sirococcus linderae* (CGMCC 3.40299) also formed a distinct, highly supported clade (MLBS = 100%, BIPP = 1.00) and was sister to *S. lanceolatae*. The strains of *S. fujianensis* (SAUCC 10881) and *S. mengyinensis* (SAUCC 10843) each clustered with their respective ex-type strains with high statistical support (MLBS = 100%, BIPP = 1.00). In addition, the four species *S. xishuangbannaensis*, *S. yunnanensis*, *S. taishanensis*, and *S. chinensis* were recovered in a poorly supported clade in our phylogenetic tree, within which they formed two species pairs: *S. xishuangbannaensis*–*S. yunnanensis* and *S. taishanensis*–*S. chinensis*. To further examine the species boundaries within these pairs, we performed a detailed comparative evaluation, as presented in the section Critical evaluation of closely related *Sirococcus* species. Results of single-gene phylogenetic analyses are provided in Suppl. material 2.

### Taxonomy

#### Sirococcus euryicola

Taxon classificationFungiDiaporthalesGnomoniaceae

Y.L. Wang, Z.X. Zhang, M.Y. Zhang sp. nov.

CAC149D1-66C4-5EA3-BC64-1F5C811FD8AA

864289

Fig. 2

##### Type.

China. • Yunnan Province: Mopanshan National Forest Park, 23°10'01"N, 101°09'37"E, ca. 2,123 m a.s.l., isolated from healthy leaves of *Eurya japonica*, 13 May 2024, coll. Y.L. Wang (holotype: dried culture, HSAUP 8416; ex-holotype culture CGMCC 3.40298).

**Figure 2.**
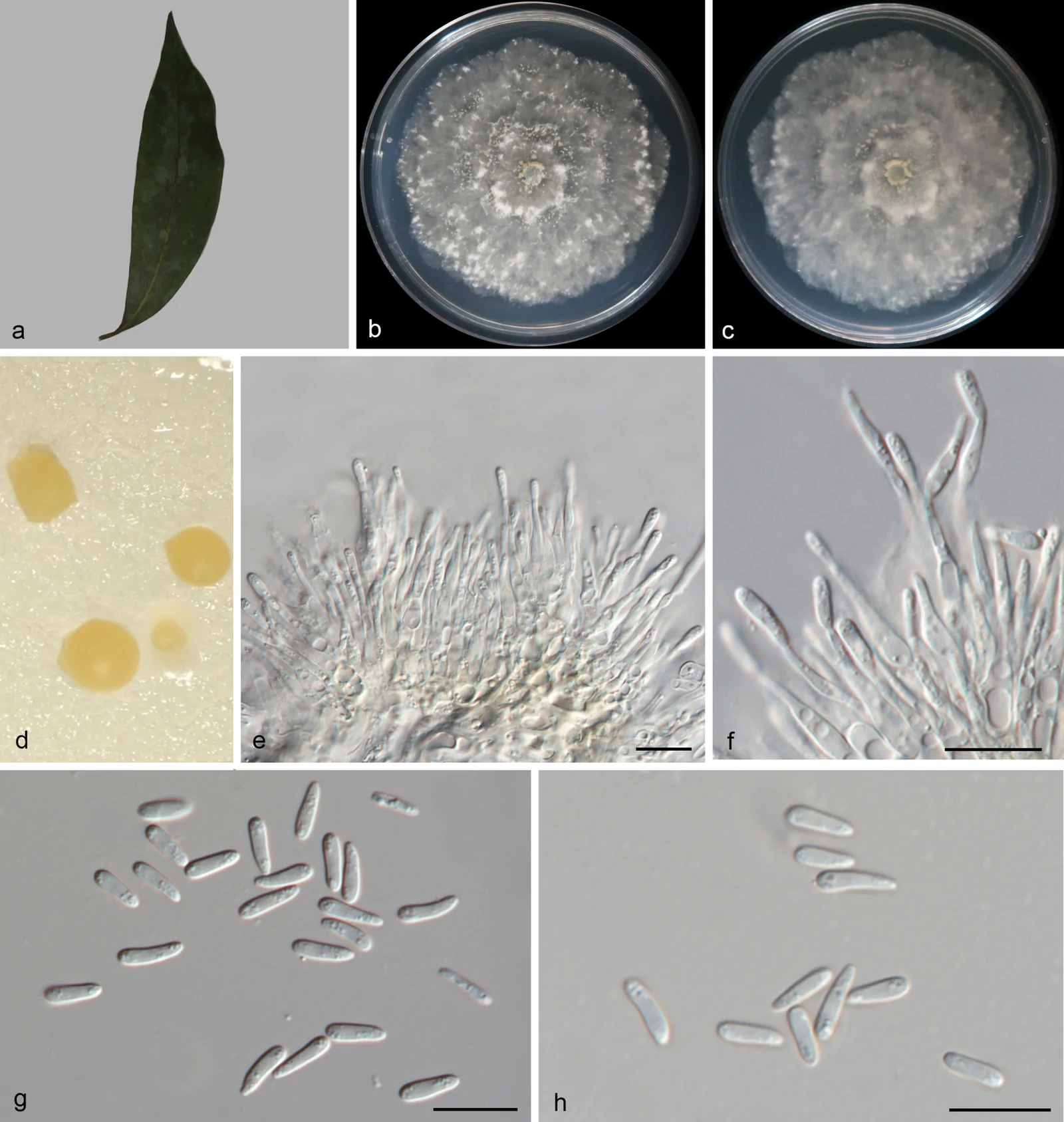
*Sirococcus euryicola* sp. nov. (CGMCC 3.40298, ex-type). **a**. Host-plant leaf; **b, c**. Obverse and reverse sides of colony after seven days on PDA; **d**. Colony overview; **e, f**. Conidiogenous cells and conidia; **g, h**. Conidia. Scale bars: 10 μm (**e–h**).

##### Etymology.

The specific epithet euryicola refers to the host plant *Eurya japonica*.

##### Description.

Sexual morph not observed. Asexual morph: pycnidial conidiomata solitary, yellowish-brown, producing hyaline conidial masses after 10 d of incubation on WA at 25 °C in darkness. Conidiophores hyaline, smooth, indistinct, mostly reduced to conidiogenous cells. Conidiogenous cells phialidic, hyaline, smooth, cylindrical to ampulliform, 6.4–15.3 × 1.2–2.3 μm. Conidia hyaline, smooth, guttulate, obovoid to oval-fusoid, straight or slightly curved, base acute, (5.3–)6.2–7.8(–9.4) × (1.4–)1.6–2.0(–2.2) μm, mean ± SD = 6.99 ± 0.82 × 1.79 ± 0.16 μm, n = 46.

##### Culture characteristics.

Colonies on PDA attaining 73–74 mm diam. after 7 d at 25 °C in darkness, petaloid, greyish-white, moderate aerial mycelium; margin undulate; reverse concolorous; no diffusible pigments.

##### Additional materials examined.

China. • Yunnan Province: Mopanshan National Forest Park, 23°10'01"N, 101°09'37"E, ca. 2,123 m a.s.l., on healthy leaves of *Eurya japonica*, 13 May 2024, coll. Y.L. Wang, culture SAUCC 8420.

##### Notes.

Multilocus phylogenetic analysis based on the combined ITS, *tef1*, and *tub2* sequence dataset reveals that *S. euryicola* forms a highly supported monophyletic clade (MLBS = 100%, BIPP = 1.00) within *Sirococcus* and is sister to *S. mengyinensis* (Fig. 1). Pairwise sequence comparisons show that *S. euryicola* differs from *S. mengyinensis* (SAUCC MY0293, ex-type) by 1.9% (9/485), 11.5% (69/600), and 6.8% (31/453) nucleotide divergence in the ITS, *tef1*, and *tub2* regions, respectively. *Sirococcus euryicola* is morphologically distinguished from *S. mengyinensis* by its longer, more slender and predominantly obovoid conidia ((5.3–)6.2–7.8(–9.4) × (1.4–)1.6–2.0(–2.2) μm), whereas conidia of *S. mengyinensis* are mostly cylindrical (4.5–6.5 × 1.8–2.8 μm) (Wang et al. 2022). This species is currently known only from healthy leaves of *Eurya japonica* in Yunnan Province, China.

#### Sirococcus fujianensis

Taxon classificationFungiDiaporthalesGnomoniaceae

(Zhao X. Zhang & X.G. Zhang) Ning Jiang, in Jiang, Wijayawardene, Xue & Li, IMA Fungus 17: 13 (2026)

E553DA17-713A-5BDB-9F68-8395F45BBE11

Fig. 3

##### Ecology.

Isolated from diseased leaf tissues of *Camellia sinensis* (*Theaceae*). New host-record.

**Figure 3.**
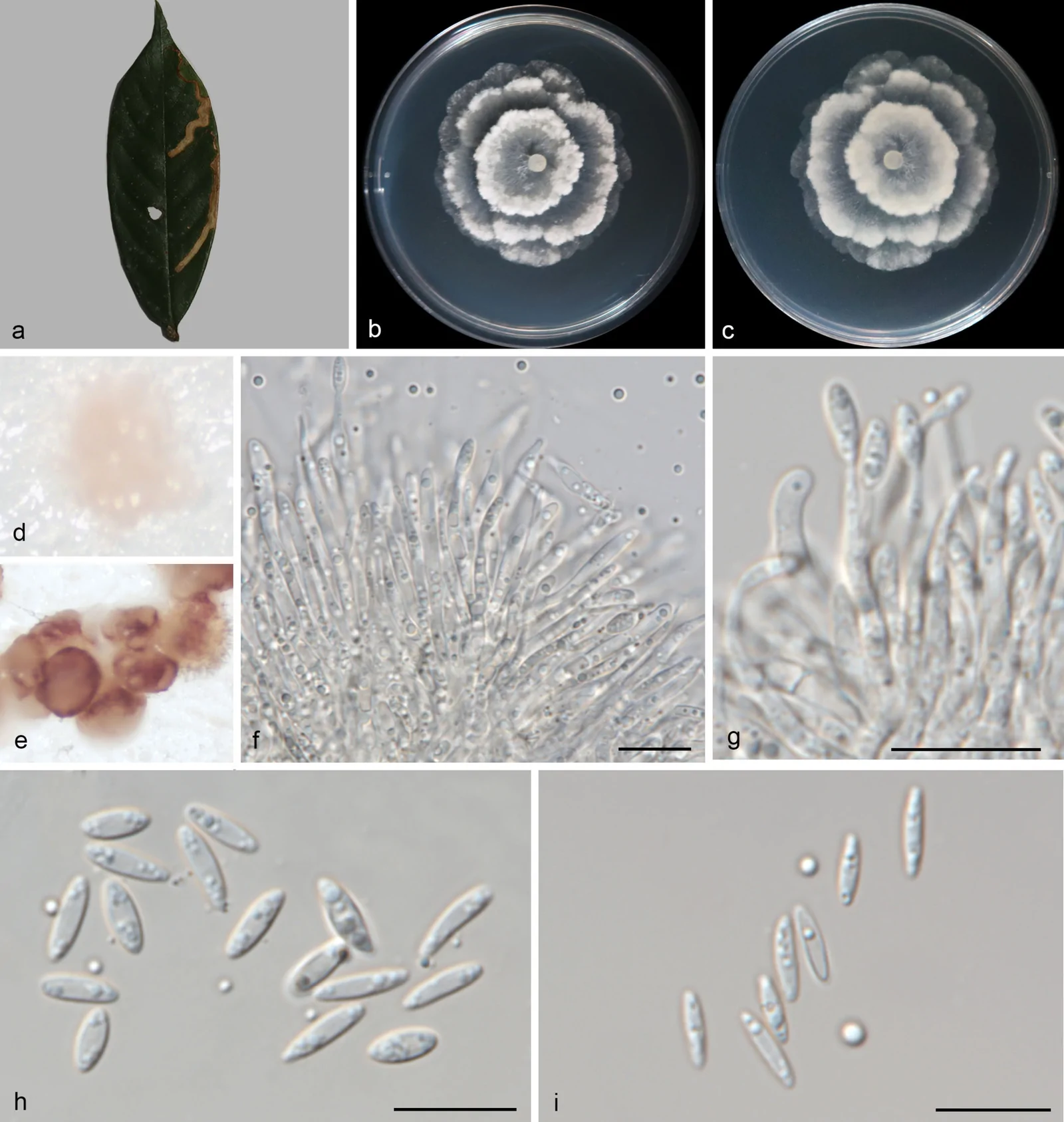
*Sirococcus fujianensis* (SAUCC 10881). **a**. Host-plant leaf; **b, c**. Obverse and reverse sides of colony after seven days on PDA; **d, e**. Colony overview; **f, g**. Conidiogenous cells and conidia; **h, i**. Conidia. Scale bars: 10 μm (**f–i**).

##### Description.

Sexual morph not observed. Asexual morph: Pycnidial conidiomata, solitary to aggregated, globose to pulvinate, reddish-brown, exuding creamy pale-pink conidial masses after 10 d of incubation at 25 °C in darkness. Conidiophores hyaline, smooth, indistinct, often reduced to conidiogenous cells. Conidiogenous cells phialidic, hyaline, smooth, cylindrical to ampulliform, attenuate towards the apex, 15.7–22.7 × 1.5–2.0 μm. Conidia hyaline, smooth, multi-guttulate, obovoid to oval-fusoid, straight or slightly curved, base truncate, (4.9–)5.7–7.4(–8.1) × (1.7–)1.8–2.3(–2.7) μm, mean ± SD = 6.54 ± 0.81 × 2.09 ± 0.25 μm, n = 33.

##### Culture characteristics.

Colonies on PDA attaining 54–55 mm diam. after 7 d at 25 °C in darkness, spreading, petaloid, greyish-white, with moderate aerial mycelium; margin undulate; reverse concolorous.

##### Additional materials examined.

China. • Fujian Province: Wuyishan City, Rare and Precious Botanical Garden, 27°07'47"N, 117°07'17"E; ca. 724 m a.s.l., on leaves of *Camellia sinensis*, 19 October 2024, coll. Y.L. Wang, culture: SAUCC 10881.

##### Notes.

Multilocus phylogenetic analysis based on the combined ITS, *tef1*, and *tub2* dataset shows that the strain obtained in this study (SAUCC 10881) clusters with *Sirococcus fujianensis* with high statistical support (MLBS = 100%, BIPP = 1.00) (Fig. 1). Pairwise sequence comparisons reveal only negligible intraspecific nucleotide differences: 0% divergence in the ITS region, 0.64% (4/621) in *tef1*, and 0.23% (1/441) in *tub2*, which are far below the commonly accepted interspecific divergence thresholds for *Sirococcus* (≥ 1% for ITS, ≥ 3% for *tef1*, ≥ 1% for *tub2*). Morphologically, the conidial dimensions of the strain in this study (SAUCC 10881; (4.9–)5.7–7.4(–8.1) × (1.7–)1.8–2.3(–2.7) μm) completely overlap with those of the ex-type strain CGMCC 3.28229 (4.8–7.5 × 1.7–2.5 μm), and no distinct morphological differences were observed in colony characteristics or conidiogenous cell morphology.

*Sirococcus fujianensis* was originally described as an endophytic fungus isolated from leaves of *Castanopsis chinensis* in Fujian Province, China (Zhang et al. 2025). Prior to this study, it was known only from its type host *Castanopsis chinensis* (*Fagaceae*, *Castanopsis*). This study presents the first record of *S. fujianensis* on *Camellia sinensis* (*Theaceae*), extending its known host range to *Theaceae*. This finding indicates that *S. fujianensis* has broader host adaptability than previously documented. Based on congruent results of morphological observations and multi-locus phylogenetic analysis, we report *Sirococcus fujianensis* as a new host record on *Camellia sinensis* in Fujian Province, China.

#### Sirococcus linderae

Taxon classificationFungiDiaporthalesGnomoniaceae

Y.L. Wang, Z.X. Dong, Y.X. Shang sp. nov.

B77DFDB2-624C-5447-B831-FDA17C30D23B

864292

Fig. 4

##### Type.

China. • Fujian Province: Wuyishan Da’anyuan Ecological Tourism Scenic Area, 27°09'13"N, 117°08'56"E; ca. 497 m a.s.l., on healthy leaves of *Lindera aggregata*, 20 October 2024, coll. Y.L. Wang (holotype: dried culture, HSAUP 11003; ex-holotype culture CGMCC 3.40299).

**Figure 4.**
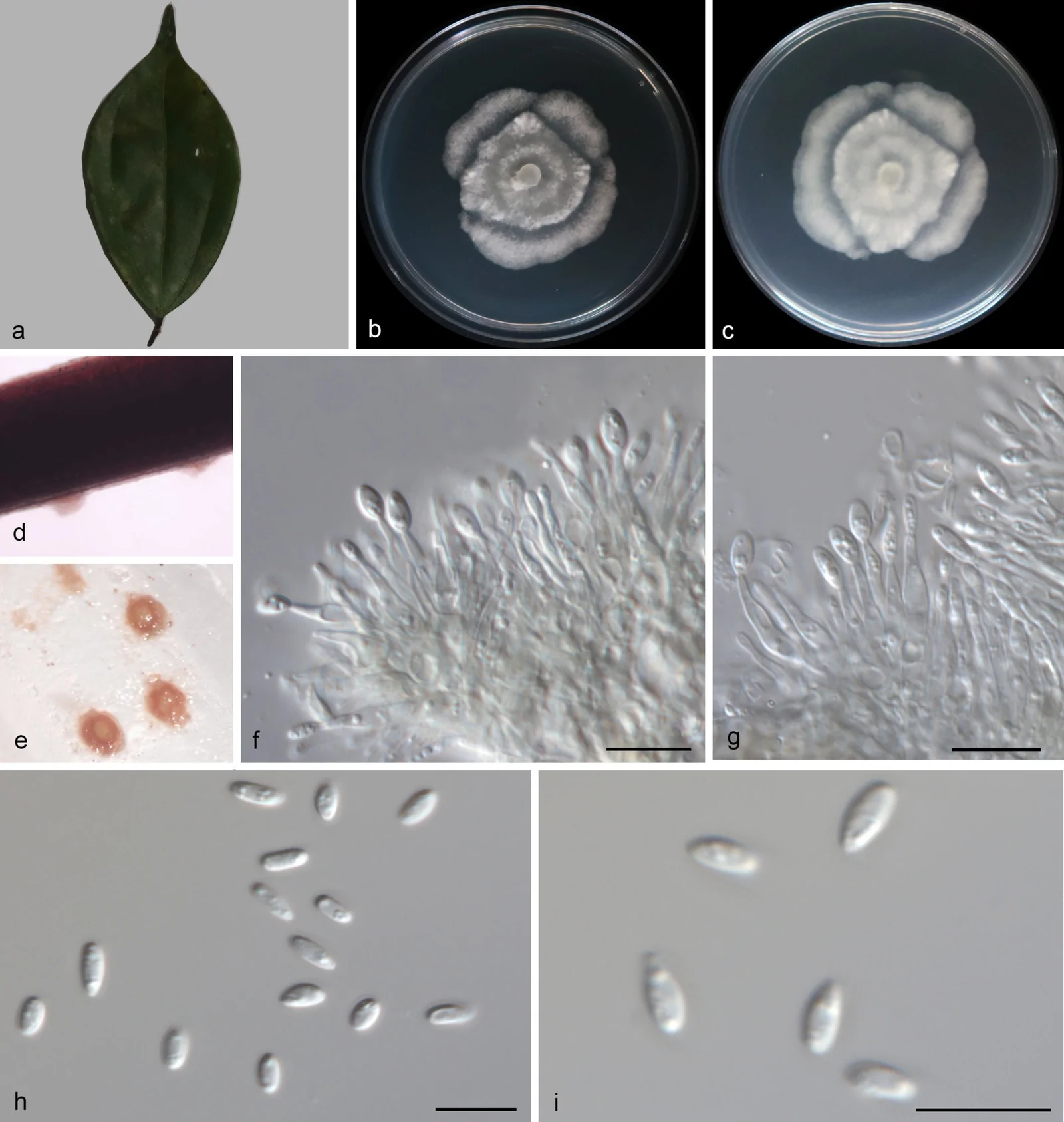
*Sirococcus linderae* sp. nov. (CGMCC 3.40299, ex-type). **a**. Host-plant leaf; **b, c**. Obverse and reverse sides of colony after 7 d on PDA; **d, e**. Colony overview; **f, g**. Conidiogenous cells and conidia; **h, i**. Conidia. Scale bars: 10 μm (**f–i**).

##### Etymology.

The specific epithet linderae refers to the host plant *Lindera aggregata*.

##### Description.

Sexual morph not observed. Asexual morph: pycnidial conidiomata solitary, globose to pulvinate, reddish-brown; hyaline conidial masses exuded after 14 d on WA at 25 °C in darkness. Conidiophores hyaline, smooth, mostly reduced to conidiogenous cells. Conidiogenous cells hyaline, smooth, cylindrical to ampulliform, 9.4–13.4 × 1.2–2.2 μm. Conidia hyaline, smooth, guttulate, obovoid to oval fusoid, base truncate, (3.8–)4.8–6.0(–6.5) × (2.0–)2.2–2.6(–2.8) μm, mean ± SD = 5.44 ± 0.61 × 2.42 ± 0.20 μm, n = 33.

##### Culture characteristics.

Colonies on PDA attaining 45–50 mm diam. after 7 d at 25 °C in darkness; petaloid, greyish-white, moderate aerial mycelium; margin undulate; reverse concolorous.

##### Additional materials examined.

China. • Fujian Province: Wuyishan Da’anyuan Ecological Tourism Scenic Area, 27°09'13"N, 117°08'56"E; ca. 497 m a.s.l., on healthy leaves of *Lindera aggregata*, 20 October 2024, coll. Y.L. Wang, culture SAUCC 11015.

##### Notes.

Multilocus phylogenetic analysis based on the combined ITS, *tef1*, and *tub2* sequence dataset revealed that *S. linderae* forms a highly supported monophyletic clade (MLBS = 100%, BIPP = 1.00) within *Sirococcus*, sister to *S. lanceolatae* (Fig. 1). Pairwise sequence comparisons show that *S. linderae* differs from the ex-type strain of *S. lanceolatae* (CGMCC 3.28232) by 8.5% (51/597), 39.2% (245/625), and 28.1% (108/384) nucleotide differences in the ITS, *tef1*, and *tub2* loci, respectively. Morphologically, *S. linderae* can be readily distinguished from *S. lanceolatae* by its characteristic petaloid colonies on PDA (Zhang et al. 2025). In addition, marked differences in conidial dimensions further separate the two species: conidia of *S. linderae* measure (3.8–)4.8–6.0(–6.5) × (2.0–)2.2–2.6(–2.8) μm, whereas those of *S. lanceolatae* are 5.5–8.5 × 1.3–1.8 μm. Based on congruent morphological and multilocus phylogenetic evidence, we formally describe *Sirococcus linderae* as a new species.

#### Sirococcus mengyinensis

Taxon classificationFungiDiaporthalesGnomoniaceae

(Shi Wang, Zhao X. Zhang, X.Y. Liu & X.G. Zhang) Ning Jiang, in Jiang, Wijayawardene, Xue & Li, IMA Fungus 17: 14 (2026)

9A8D03EB-0922-5523-92F6-8468EEC96616

Fig. 5

##### Distribution.

China (Shandong Province). New geographical record.

**Figure 5.**
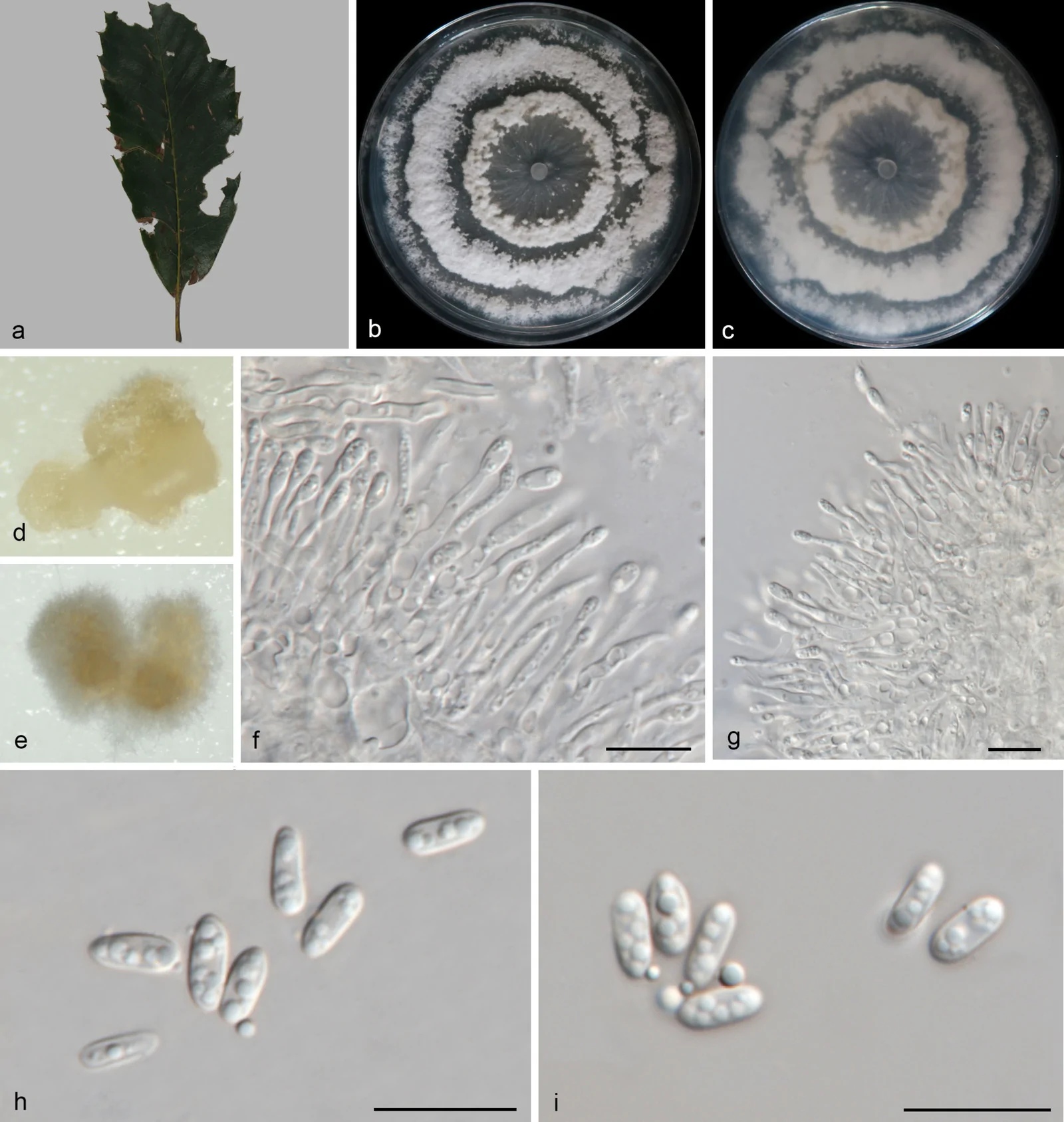
*Sirococcus mengyinensis* (SAUCC 10843). **a**. Host-plant leaf; **b, c**. Obverse and reverse sides of colony after 7 d on PDA; **d, e**. Colony overview; **f, g**. Conidiogenous cells and conidia; **h, i**. Conidia. Scale bars: 10 μm (**f–i**).

##### Ecology.

Isolated from diseased leaves of *Castanea mollissima* (*Fagaceae*).

##### Description.

Sexual morph not observed. Asexual morph: pycnidial conidiomata aggregated or solitary, erumpent, light-yellow, exuding hyaline conidial masses after 10 d of incubation at 25 °C in darkness. Conidiophores hyaline, smooth, indistinct, often reduced to conidiogenous cells. Conidiogenous cells phialidic, hyaline, smooth, cylindrical to ampulliform, attenuate towards the apex, 11.4–21.2 × 0.9–1.6 μm. Conidia hyaline, smooth, multi-guttulate, ellipsoid to oblong, straight or slightly curved, base truncate, (5.2–)5.4–6.5(–7.5) × (2.0–)2.1–2.5(–2.8) μm, mean ± SD = 5.96 ± 0.54 × 2.30 ± 0.16 μm, n = 34.

##### Culture characteristics.

Colonies on PDA at 25 °C in darkness, attaining 83–85 mm diam. after seven days, spreading, petaloid, greyish-white, with moderate aerial mycelium; margin undulate, circular in outline; reverse concolorous.

##### Additional materials examined.

China. • Shandong Province: Taishan District, Shaling Village, 36°02'36"N, 117°01'15"E; ca. 155 m a.s.l., on leaves of *Castanea mollissima*, 9 October 2024, coll. Y.L. Wang, culture: SAUCC 10843.

##### Notes.

Multilocus phylogenetic analysis based on the combined ITS, *tef1*, and *tub2* dataset shows that the strain obtained in this study (SAUCC 10843) clusters with *Sirococcus mengyinensis* with high statistical support (MLBS = 100%, BIPP = 1.00) (Fig. 1). Pairwise sequence comparisons reveal only minor intraspecific nucleotide differences: 0% divergence in the ITS region, 2.86% (18/629) in *tef1*, and 0.45% (2/442) in *tub2*, all of which are well below the interspecific variation thresholds reported for *Sirococcus* (≥ 1% for ITS, ≥ 3% for *tef1*, ≥ 1% for *tub2*). Morphologically, the conidial dimensions of the strain in this study (SAUCC 10843; (5.2–)5.4–6.5(–7.5) × (2.0–)2.1–2.5(–2.8) μm) overlap substantially with those of the ex-type strain SAUCC MY0293 (4.5–6.5 × 1.8–2.8 μm), and no stable morphological differences were observed in colony characteristics or conidiogenous cell morphology.

*Sirococcus mengyinensis* was previously known only from Mengyin, Shandong Province. This study provides the first record of *S. mengyinensis* in Tai’an City, extending its known distribution range approximately 110 km northwestward within Shandong Province. Notably, *S. mengyinensis* was isolated from symptomatic chestnut (*Castanea mollissima*) leaves both in this study and in Wang et al. (2022), suggesting that this species is associated with chestnut leaf lesions. However, its precise pathogenic potential and ecological niche remain to be further elucidated. Based on the congruent results of morphological observations and multilocus phylogenetic analysis, we report *Sirococcus mengyinensis* as a new geographical record for Tai’an City, Shandong Province, China.

### Key to the species of *Sirococcus*

Together with the two new species described in this study, the *Sirococcus* currently comprises a worldwide total of approximately 40 species. In order to facilitate identification in the future, a key to the species of *Sirococcus* is provided herein. Characteristics adopted in the key include perithecia, asci, ascospores, septation, conidiomata, conidiogenous cells, conidia, and host association.

**Table:** 

1	Sexual morph known	**2**
–	Sexual morph unknown or not observed	**16**
2	Host *Onagraceae* (*Chamaenerion angustifolium*)	***S. racemulus***
–	Host not *Onagraceae*	**3**
3	Host *Fagaceae*	**4**
–	Host *Rosaceae*	**7**
4	Ascospores mean width ≥ 4 μm, distinctly constricted at septum	**5**
–	Ascospores mean width < 4 μm	**6**
5	Host *Quercus falcata*; ascospores 5–11 × 2–5.5 μm	***S. clavulatus***
6	Host *Quercus alba*; ascospores 8–11 × 3–4 μm	***S. paraclavulatus***
–	Host *Castanea* / *Quercus ilex*; ascospores 4–17 × 1–3 μm, constricted at septum; pathogenic	***S. smithogilvyi***
7	Host *Rubus*	**8**
–	Host not *Rubus*	**11**
8	Ascospores mean length ≥ 10.5 μm	**9**
–	Ascospores mean length < 10.5 μm	**10**
9	Ascospores 10–13 × 2–3 μm, mean 11 × 2.5; septum located at 36%; host *Rubus chamaemorus*	***S. chamaemori***
10	Host *Rubus parviflorus* / *R. pedatus*; ascospores 9–11 × 2–4 μm	***S. alderdunensis***
–	Host various *Rubus*; ascospores 6.5–11 × 2–3.5 μm, mean 9 × 3; with 1–4 guttules	***S. idaeicola***
11	Host *Comarum*	***S. comari***
–	Host not *Comarum*	**12**
12	Host *Fragaria*	***S. fragariae***
–	Host not *Fragaria*	**13**
13	Host *Agrimonia*	***S. guttulatus***
–	Host not *Agrimonia*	**14**
14	Host *Spiraea*	***S. macounii***
–	Host not *Spiraea*	**15**
15	Host *Potentilla*	***S. occultus***
–	Host *Sanguisorba*	***S. sanguisorbae***
16	Conidia 1-septate or 0–1-septate	**17**
–	Conidia strictly 0-septate	**21**
17	Conidia 0–1-septate, 10–16.1 × 3.1–4.1 μm, slightly constricted at septum; host *Castanopsis hainanensis*	***S. rossmaniae***
–	Conidia strictly 1-septate	**18**
18	Host *Pinaceae* / *Cupressaceae*	**19**
19	Conidiomatal wall textura angularis, outer wall cells 2–8 μm; conidiogenous cells ampulliform; conidia 8.6–14 × 2.1–4 μm, L/W 3.6; host *Picea*	***S. piceicola***
–	Conidiomatal wall textura intricata, outer wall cells 2–4 μm; conidiogenous cells cylindrical	**20**
20	Conidia 10.2–15.8 × 2.2–3.5 μm, L/W 4.5; host *Pinaceae* and *Cupressaceae* (*Cedrus deodara*)	***S. conigenus***
–	Conidia 10.6–15.1 × 2.5–4.0 μm, L/W 3.8; host *Tsuga* spp. and *Cedrus* spp.	***S. tsugae***
21	Host *Fagaceae*	**22**
–	Host not *Fagaceae*	**37**
22	Conidia Lmax > 10 μm	**23**
–	Conidia Lmax ≤ 10 μm	**25**
23	Conidia 14–20 × 4–5.5 μm; conidiomata 115–250 μm; host *Castanopsis fissa*	***S. xunwuensis***
–	Conidia Lmax 10–12.6 μm	**24**
24	Conidia 9–12.6 × 2.8–4.5 μm; conidiomata acervular, 250–450 μm, red-brown; host various *Fagaceae*	***S. fagacearum***
–	Conidia 7.3–12.2 × 3.3–4.2 μm; conidiomata pycnidial, 100–300 μm, light brown; host *Castanopsis hainanensis*	***S. hainanensis***
25	Conidia Lmax < 6.0 μm	**26**
–	Conidia Lmax ≥ 6.0 μm	**30**
26	Conidia base circular; conidiomata pycnothyria; host *Lithocarpus fohaiensis*	***S. lithocarpi***
–	Conidia base truncate	**27**
27	Conidiomata dark brown, 150–600 μm; conidia 4.3–5.2 × 1.4–2.0 μm, constricted at middle; host *Castanopsis fargesii*	***S. guangdongensis***
–	Conidiomata not as above	**28**
28	Conidia width ≤ 2.0 μm; conidiomata pycnothyria, light yellow; host *Castanea mollissima*	***S. yunnanensis***
–	Conidia width > 2.0 μm	**29**
29	Conidia 4.3–5.4 × 1.8–2.6 μm; conidiomata 300–700 μm; host *Castanopsis hystrix*	***S. castanopsidis***
–	Conidia 4.3–5.9 × 1.9–2.7 μm; conidiomata 250–650 μm; host *Castanopsis hystrix* / *Quercus serrata* / *Castanopsis carlesii*	***S. silvicola***
30	Conidia Lmax ≥ 9.0 μm	**31**
–	Conidia Lmax < 9.0 μm	**32**
31	Conidia 6–9 × 2.2–3.5 μm; conidiomata 400–1000 × 500–1500 μm, orange tendrils; pathogenic; host *Castanea mollissima*	***S. chinensis***
–	Conidia 5.5–9 × 2–3 μm; conidiomata 200–450 μm, hazel/scarlet; host *Castanopsis carlesii*	***S. flavus***
32	Conidia width upper limit ≤ 2.0 μm	**33**
–	Conidia width upper limit > 2.0 μm	**34**
33	Host *Castanopsis chinensis*	***S. diaoluoshanensis***
–	Host *Castanea mollissima*	***S. taishanensis***
34	Host *Castanopsis chinensis*	***S. fujianensis***
–	Host *Castanea mollissima*	**35**
35	Conidiomata 100–150 μm; conidia 5–7 × 1.6–2.6 μm	***S. xishuangbannaensis***
–	Conidiomata larger than 150 μm or pycnothyria	**36**
36	Conidiomata 150–950 μm, light orange to white; conidia 5–8 × 2–3.5 μm; fruit rot	***S. daii***
–	Conidiomata pycnothyria, light brown; conidia 4.5–6.5 × 1.8–2.8 μm	***S. mengyinensis***
37	Host *Theaceae*	**38**
–	Host not *Theaceae*	**39**
38	Host *Eurya nitida*; conidiomata black, 100–150 μm; conidia 7–9.5 × 1.8–2.2 μm	***S. euryae***
–	Host *Eurya japonica*; conidiomata yellowish-brown, solitary; conidia 5.3–9.4 × 1.4–2.2 μm	***S. euryicola* sp. nov**.
39	Host *Lauraceae*	**40**
–	Host not *Lauraceae*	**41**
40	Conidia 3.8–6.5 × 2.0–2.8 μm; conidiomata reddish-brown; host *Lindera aggregata*	***S. linderae* sp. nov**.
–	Conidia 5.5–8.5 × 1.3–1.8 μm; conidiomata black, 100–150 μm; host *Phoebe lanceolata*	***S. lanceolatae***
41	Host *Annonaceae*	***S. annonae***
–	Host not *Annonaceae*	**42**
42	Host *Juglandaceae*	***S. juglandis***
–	Host not *Juglandaceae*	**43**
43	Host *Melastomataceae*	***S. melastomatis***
–	Host not *Melastomataceae*	**44**
44	Host *Rosaceae*	***S. rosae***
–	Host unknown / saprophytic	**45**
45	Conidia 8–11 × 2–2.5 μm, subcylindrical; conidiomata multi-locular eustromatic	***S. angolensis***
–	Conidia 4–5.4 × 1.3–2.2 μm, ellipsoid; conidiomata 100–150 μm	***S. saprophyticus***

### Critical evaluation of closely related *Sirococcus* species

Phylogenetic reconstruction recovered *Sirococcus xishuangbannaensis* and *Sirococcus yunnanensis* as one pair and *Sirococcus taishanensis* and *Sirococcus chinensis* as another pair within a poorly supported clade (Fig. 1). To resolve the resulting taxonomic uncertainties among these closely related chestnut-associated taxa, we performed a critical conspecificity evaluation of the two species pairs. None of these four species were isolated in the present study. We retrieved ITS, *tef1*, and *tub2* sequences of their ex-type strains from GenBank. Sequences were aligned and trimmed using the same criteria adopted for our multilocus phylogenetic dataset, and pairwise p-distances were calculated. Morphological and ecological comparisons were based on their original published protologues.

For *S. xishuangbannaensis* and *S. yunnanensis*, pairwise sequence divergences were 8/502 for ITS, 11/625 for *tef1*, and 3/453 for *tub2*. The divergence of the ITS locus exceeds the interspecific threshold for *Sirococcus*, while variations in *tef1* and *tub2* fall within the typical intraspecific range. Morphologically, the two taxa can be reliably distinguished by conidial dimensions: conidia of *S. xishuangbannaensis* (5–7 × 1.6–2.6 μm) are distinctly larger than those of *S. yunnanensis* (4.1–5.5 × 1.3–2.0 μm), with only limited size overlap (Wang et al. 2022; Zhang et al. 2025). Given this stable phenotypic differentiation, both taxa are maintained as distinct species, and no synonymisation is proposed here.

For *S. taishanensis* and *S. chinensis*, pairwise comparisons revealed very low genetic divergence: 2/506 in ITS, 3/569 in *tef1*, and 0/442 in *tub2*. All values are well below the interspecific thresholds defined for *Sirococcus*. Nevertheless, multiple consistent phenotypic and ecological differences are documented in their original descriptions. *Sirococcus taishanensis* was isolated from diseased leaves of *Castanea mollissima*, forms pycnidial conidiomata and smaller conidia (3.8–6.5 × 1.2–2.0 μm), and produces greyish-sepia to grey-creamy colonies on PDA (Zhang et al. 2025). In contrast, *S. chinensis* was obtained from stem- and branch-canker tissues of *C. mollissima*. It develops large pseudostromatic conidiomata that exude orange conidial tendrils, bears markedly larger conidia ((6.0–)6.5–8.5(–9.0) × (2.2–)2.7–3(–3.5) μm, and forms PDA colonies with a dark-brown centre fading to pale-grey at the margin (Jiang et al. 2020). Such phenotypic divergence with minimal nuclear-sequence variation has been reported within the *Diaporthales* (Zhang et al. 2025). Owing to the lack of additional intermediate isolates and experimental evidence confirming whether these morphological differences result from phenotypic plasticity, we refrain from formal synonymisation at this stage and retain both as valid species. We highlight that further sampling of additional isolates is required to better resolve their species boundaries. This assessment does not modify any taxonomic conclusions reported elsewhere in the present study.

## Discussion

In this study, we systematically investigated the diversity of *Sirococcus* based on diseased and healthy leaves collected from Fujian, Shandong, and Yunnan provinces, China. More than half of the isolates were obtained from hosts in *Fagaceae*, whereas the remaining isolates were recovered from hosts in *Lauraceae*, *Melastomataceae*, *Rosaceae*, and *Theaceae*. Based on morphological observations and multilocus phylogenetic analysis (ITS, *tef1*, and *tub2*), four taxa were recognised: two new species (*Sirococcus euryicola* sp. nov. and *Sirococcus linderae* sp. nov.), a new host record for *S. fujianensis*, and a new geographical record for *S. mengyinensis*. Previous records of *Sirococcus* in China were largely limited to pathogenic taxa associated with Chinese Chestnut (*Castanea mollissima*) (Jiang and Tian 2019; Jiang et al. 2020). This study substantially enriches the documented species diversity of *Sirococcus* in China and advances our understanding of its host range and geographical distribution.

Despite the diversity documented here, species delimitation within *Sirococcus* on *Castanea mollissima* remains challenging, as revealed by our phylogenetic analyses. In the combined-locus tree, the clade comprising *S. xishuangbannaensis*, *S. yunnanensis*, *S. taishanensis*, and *S. chinensis* received low branch support (Fig. 1). Given this low support, we performed a critical assessment of conspecificity for the two species pairs within this clade, *S. xishuangbannaensis*–*S. yunnanensis* and *S. taishanensis*–*S. chinensis*. Specifically, phenotypic divergence is accompanied by moderate genetic differentiation between *S. xishuangbannaensis* and *S. yunnanensis*. By contrast, *S. taishanensis* and *S. chinensis* exhibit stable phenotypic and ecological differences despite near-identical sequences at the ITS, *tef1*, and *tub2* regions. Such decoupling of morphological and ecological variation from nuclear gene divergence has been reported in fungi of the order *Diaporthales* (Zhang et al. 2025). This indicates that multilocus DNA data alone cannot reliably delimit species boundaries; phenotypic and ecological evidence should also be considered in taxonomic decisions. Broader sampling of field-collected isolates and experiments addressing phenotypic plasticity are needed to resolve species boundaries among these chestnut-inhabiting fungi.

Beyond species delimitation, our data, together with recent taxonomic revisions (Jiang et al. 2026), demonstrate that *Sirococcus* is not restricted to a narrow set of hosts as previously assumed. The genus now encompasses species associated with at least 10 plant families, including *Fagaceae*, *Rosaceae*, *Onagraceae*, *Pinaceae*, *Cupressaceae*, *Theaceae*, *Lauraceae*, *Annonaceae*, *Juglandaceae*, and *Melastomataceae* (Sogonov et al. 2008; Walker et al. 2010; Crous et al. 2018; Jiang et al. 2021b; Wang et al. 2022; Zhang et al. 2025). For instance, *S. fujianensis* was originally described from *Castanopsis chinensis* (*Fagaceae*) but is here reported on *Camellia sinensis* (*Theaceae*), representing a host jump across distantly related families. Similarly, *S. smithogilvyi* occurs on *Castanea* and *Quercus (Fagaceae)*, while *S. conigenus* and *S. tsugae* infect *Pinaceae* and *Cupressaceae* (Rossman et al. 2008). In contrast, some species exhibit strict host specificity, such as *S. chamaemori* on *Rubus chamaemorus* (*Rosaceae*) and *S. racemulus* on *Chamaenerion angustifolium* (*Onagraceae*) (Sogonov et al. 2008). This continuum from specialists to generalists suggests that host specificity in *Sirococcus* is species-specific rather than genus-wide. Ecologically, many *Sirococcus* species are endophytic or latent pathogens, as demonstrated by isolations from healthy leaves of *Eurya japonica* (*S. euryicola*), *Lindera aggregata* (*S. linderae*), *Juglans regia* (*S. juglandis*), and *Melastoma candidum* (*S. melastomatis*) (Zhang et al. 2025; present study). Others are primary pathogens causing shoot blight, leaf spots, or nut rot (Rossman et al. 2008; Jiang and Tian 2019; Li et al. 2025). The recent discovery of the teleomorph of *S. daii* on overwintered leaf litter and its endophytic phase in healthy flowers and nuts further highlights a hemibiotrophic life cycle with a saprobic overwintering stage (Jiang et al. 2026). These ecological strategies likely facilitate survival, dispersal, and host colonisation in diverse environments. The broad host range and flexible trophic modes of *Sirococcus* have significant implications for plant health, as endophytic species may become pathogenic under stress or favourable conditions, and host shifts could lead to emerging diseases in new crops. Nevertheless, the pathogenic potential of most isolates obtained in this study remains uncharacterised. Future studies should investigate the genetic basis of host specificity and the environmental factors triggering the switch from endophytism to pathogenicity.

In conclusion, this study enriches the documented species diversity of *Sirococcus* in China by describing two new species along with new host and geographical records. Further large-scale surveys covering broader geographical ranges, more host groups, and diverse plant tissues are needed to uncover cryptic diversity and clarify the ecological functions of *Sirococcus*.

## Supplementary Material

XML Treatment for Sirococcus euryicola

XML Treatment for Sirococcus fujianensis

XML Treatment for Sirococcus linderae

XML Treatment for Sirococcus mengyinensis

## Additional information

### Artificial Intelligence (AI) use

The authors accept full responsibility for the content of the manuscript, including the disclosure of any use of AI.

No AI tools were used in the preparation of this manuscript.

### Author ORCIDs

Y. Wang https://orcid.org/0009-0009-4029-2734

Q. Liu https://orcid.org/0009-0009-9545-7962

M. Zhang https://orcid.org/0009-0002-3068-7491

Z. Zhang https://orcid.org/0000-0002-4824-9716

X. Zhang https://orcid.org/0000-0001-9733-8494

Z. Li https://orcid.org/0000-0003-0583-0399

### Data availability

All of the data that support the findings of this study are available in the main text or Supplementary Information.

## Supplementary materials

Supplementary material 1 Taxonomic authorities for all fungal and plant species included in the phylogenetic analysis Data type xlsx https://binary.pensoft.net/file/1822459This dataset is made available under the Open Database License (http://opendatacommons.org/licenses/odbl/1.0/). The Open Database License (ODbL) is a license agreement intended to allow users to freely share, modify, and use this Dataset while maintaining this same freedom for others, provided that the original source and author(s) are credited. Yaling Wang, Qiyun Liu, Mengyuan Zhang, Zhaoxue Zhang, Xiuguo Zhang, Zhuang Li

Supplementary material 2 Supplementary figures Data type pdf Explanation note **fig. S1**. ITS phylogenetic tree. **fig. S2**. *tef1* phylogenetic tree. **fig. S3**. *tub2* phylogenetic tree. https://binary.pensoft.net/file/1822460This dataset is made available under the Open Database License (http://opendatacommons.org/licenses/odbl/1.0/). The Open Database License (ODbL) is a license agreement intended to allow users to freely share, modify, and use this Dataset while maintaining this same freedom for others, provided that the original source and author(s) are credited. Yaling Wang
