# Supplementary material for: Morphological and multilocus phylogenetic analysis of Sirococcus (Diaporthales, Gnomoniaceae) on woody hosts from Fujian, Shandong, and Yunnan, China: Two new species and two new records
Source: MycoKeys. 2026 Oct 27;142:239–64. doi: 10.3897/mycokeys.142.207158 (PMC13732297; doi:10.3897/mycokeys.142.207158)
Supplement: Supplementary file 1 [file mycokeys-142-239-s002.pdf]

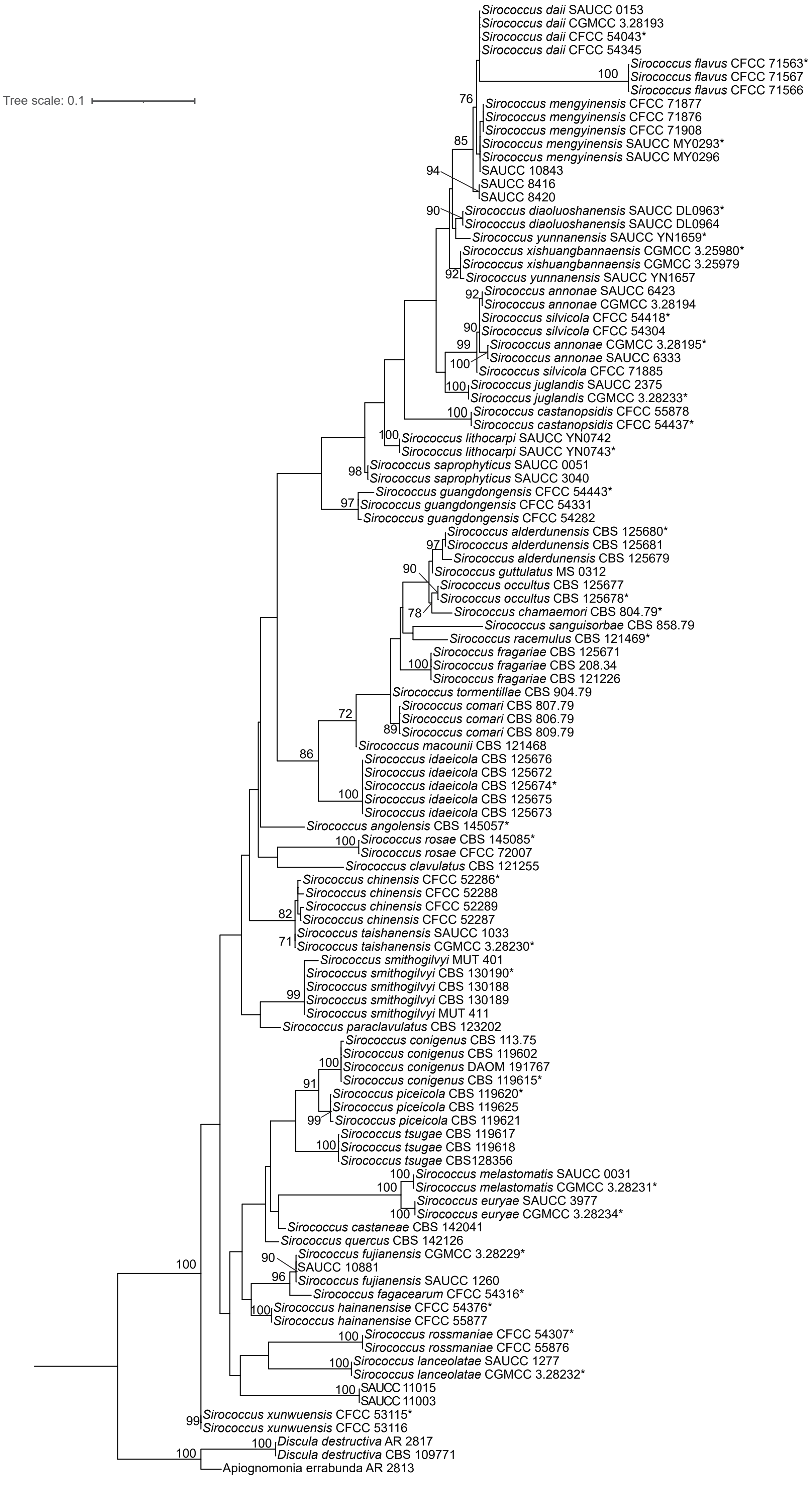

Sirococcus daii SAUCC 0153
Sirococcus daii CGMCC 3.28193
Sirococcus daii CFCC 54043*
Sirococcus daii CFCC 54345
Sirococcus flavus CFCC 71563*
100
Sirococcus flavus CFCC 71567
Sirococcus flavus CFCC 71566
Tree scale: 0.1
76
Sirococcus mengyinensis CFCC 71877
Sirococcus mengyinensis CFCC 71876
Sirococcus mengyinensis CFCC 71908
85
Sirococcus mengyinensis SAUCC MY0293*
Sirococcus mengyinensis SAUCC MY0296
SAUCC 10843
94
SAUCC 8416
SAUCC 8420
90
Sirococcus diaoluoshanensis SAUCC DL0963*
Sirococcus diaoluoshanensis SAUCC DL0964
Sirococcus yunnanensis SAUCC YN1659*
Sirococcus xishuangbannaensis CGMCC 3.25980*
Sirococcus xishuangbannaensis CGMCC 3.25979
92
Sirococcus yunnanensis SAUCC YN1657
92
Sirococcus annonae SAUCC 6423
Sirococcus annonae CGMCC 3.28194
Sirococcus silvicola CFCC 54418*
90
Sirococcus silvicola CFCC 54304
99
Sirococcus annonae CGMCC 3.28195*
Sirococcus annonae SAUCC 6333
100
Sirococcus silvicola CFCC 71885
100
Sirococcus juglandis SAUCC 2375
Sirococcus juglandis CGMCC 3.28233*
100
Sirococcus castanopsidis CFCC 55878
Sirococcus castanopsidis CFCC 54437*
100
Sirococcus lithocarpi SAUCC YN0742
Sirococcus lithocarpi SAUCC YN0743*
Sirococcus saprophyticus SAUCC 0051
98
Sirococcus saprophyticus SAUCC 3040
Sirococcus guangdongensis CFCC 54443*
97
Sirococcus guangdongensis CFCC 54331
Sirococcus guangdongensis CFCC 54282
Sirococcus alderdunensis CBS 125680*
97
Sirococcus alderdunensis CBS 125681
Sirococcus alderdunensis CBS 125679
90
Sirococcus guttulatus MS 0312
Sirococcus occultus CBS 125677
Sirococcus occultus CBS 125678*
78
Sirococcus chamaemori CBS 804.79*
Sirococcus sanguisorbae CBS 858.79
Sirococcus racemulus CBS 121469*
Sirococcus fragariae CBS 125671
100
Sirococcus fragariae CBS 208.34
Sirococcus fragariae CBS 121226
Sirococcus tormentillae CBS 904.79
72
Sirococcus comari CBS 807.79
Sirococcus comari CBS 806.79
89
Sirococcus comari CBS 809.79
Sirococcus macounii CBS 121468
86
Sirococcus idaeicola CBS 125676
Sirococcus idaeicola CBS 125672
100
Sirococcus idaeicola CBS 125674*
Sirococcus idaeicola CBS 125675
Sirococcus idaeicola CBS 125673
Sirococcus angolensis CBS 145057*
100
Sirococcus rosae CBS 145085*
Sirococcus rosae CFCC 72007
Sirococcus clavulatus CBS 121255
Sirococcus chinensis CFCC 52286*
Sirococcus chinensis CFCC 52288
Sirococcus chinensis CFCC 52289
82
Sirococcus chinensis CFCC 52287
Sirococcus taishanensis SAUCC 1033
71
Sirococcus taishanensis CGMCC 3.28230*
Sirococcus smithogilvyi MUT 401
Sirococcus smithogilvyi CBS 130190*
99
Sirococcus smithogilvyi CBS 130188
Sirococcus smithogilvyi CBS 130189
Sirococcus smithogilvyi MUT 411
Sirococcus paraclavulatus CBS 123202
Sirococcus conigenus CBS 113.75
Sirococcus conigenus CBS 119602
100
Sirococcus conigenus DAOM 191767
91
Sirococcus conigenus CBS 119615*
Sirococcus piceicola CBS 119620*
Sirococcus piceicola CBS 119625
99
Sirococcus piceicola CBS 119621
Sirococcus tsugae CBS 119617
100
Sirococcus tsugae CBS 119618
Sirococcus tsugae CBS128356
100
Sirococcus melastomatis SAUCC 0031
100
Sirococcus melastomatis CGMCC 3.28231*
Sirococcus euryae SAUCC 3977
100
Sirococcus euryae CGMCC 3.28234*
Sirococcus castaneae CBS 142041
Sirococcus quercus CBS 142126
90
Sirococcus fujianensis CGMCC 3.28229*
SAUCC 10881
100
96
Sirococcus fujianensis SAUCC 1260
Sirococcus fagacearum CFCC 54316*
100
Sirococcus hainanensise CFCC 54376*
Sirococcus hainanensise CFCC 55877
100
Sirococcus rossmaniae CFCC 54307*
Sirococcus rossmaniae CFCC 55876
100
Sirococcus lanceolatae SAUCC 1277
Sirococcus lanceolatae CGMCC 3.28232*
100
SAUCC 11015
SAUCC 11003
Sirococcus xunwuensis CFCC 53115*
99
Sirococcus xunwuensis CFCC 53116
100
100
Discula destructiva AR 2817
Discula destructiva CBS 109771
Apiognomonia errabunda AR 2813

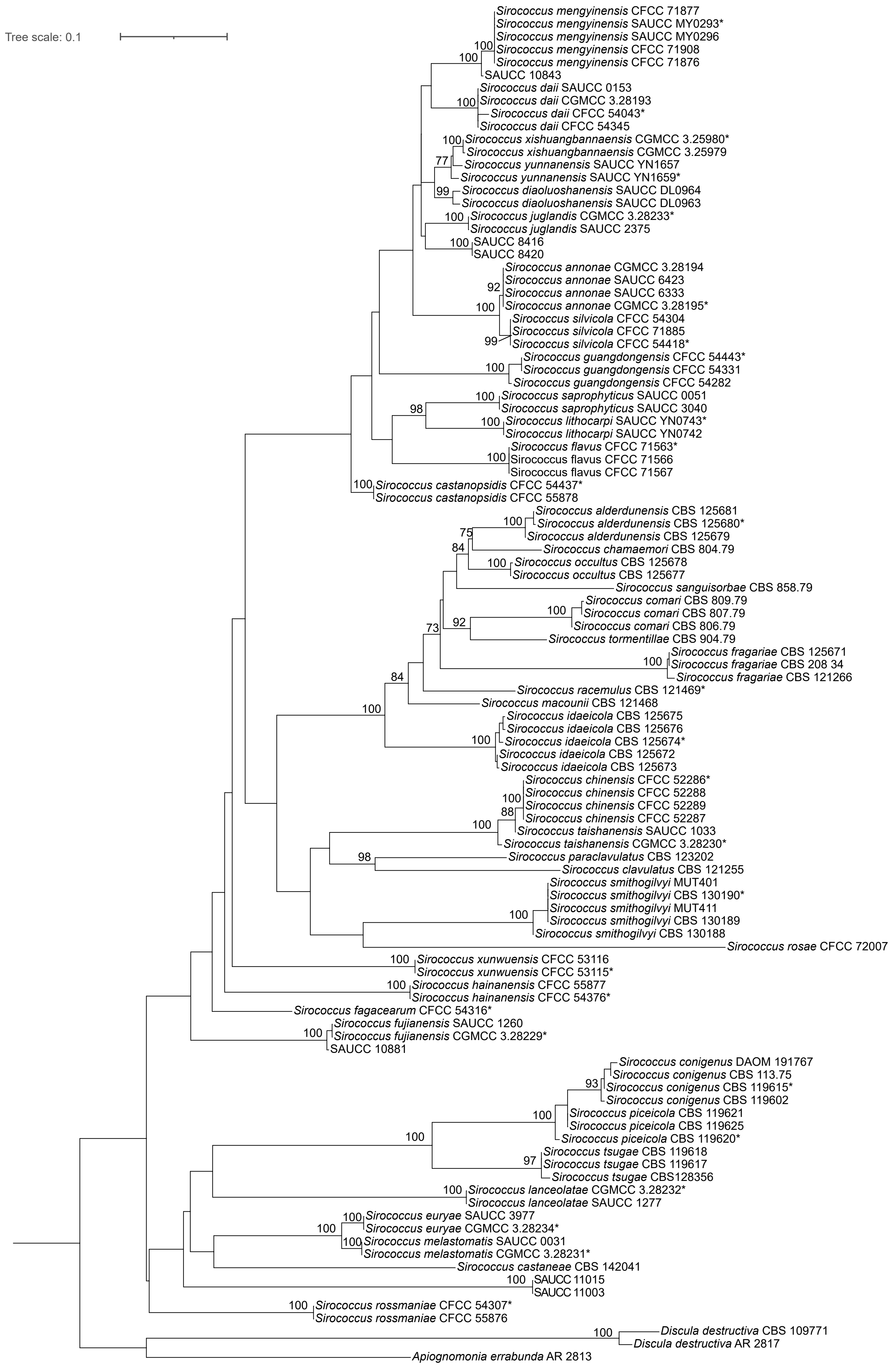
Tree scale: 0.1
Sirococcus mengyinensis CFCC 71877
Sirococcus mengyinensis SAUCC MY0293*
Sirococcus mengyinensis SAUCC MY0296
Sirococcus mengyinensis CFCC 71908
Sirococcus mengyinensis CFCC 71876
SAUCC 10843
Sirococcus daii SAUCC 0153
Sirococcus daii CGMCC 3.28193
Sirococcus daii CFCC 54043*
Sirococcus daii CFCC 54345
Sirococcus xishuangbannaensis CGMCC 3.25980*
Sirococcus xishuangbannaensis CGMCC 3.25979
Sirococcus yunnanensis SAUCC YN1657
Sirococcus yunnanensis SAUCC YN1659*
Sirococcus diaoluoshanensis SAUCC DL0964
Sirococcus diaoluoshanensis SAUCC DL0963
Sirococcus juglandis CGMCC 3.28233*
Sirococcus juglandis SAUCC 2375
SAUCC 8416
SAUCC 8420
Sirococcus annonae CGMCC 3.28194
Sirococcus annonae SAUCC 6423
Sirococcus annonae SAUCC 6333
Sirococcus annonae CGMCC 3.28195*
Sirococcus silvicola CFCC 54304
Sirococcus silvicola CFCC 71885
Sirococcus silvicola CFCC 54418*
Sirococcus guangdongensis CFCC 54443*
Sirococcus guangdongensis CFCC 54331
Sirococcus guangdongensis CFCC 54282
Sirococcus saprophyticus SAUCC 0051
Sirococcus saprophyticus SAUCC 3040
Sirococcus lithocarpi SAUCC YN0743*
Sirococcus lithocarpi SAUCC YN0742
Sirococcus flavus CFCC 71563*
Sirococcus flavus CFCC 71566
Sirococcus flavus CFCC 71567
Sirococcus castanopsidis CFCC 54437*
Sirococcus castanopsidis CFCC 55878
Sirococcus alderdunensis CBS 125681
Sirococcus alderdunensis CBS 125680*
Sirococcus alderdunensis CBS 125679
Sirococcus chamaemori CBS 804.79
Sirococcus occultus CBS 125678
Sirococcus occultus CBS 125677
Sirococcus sanguisorbae CBS 858.79
Sirococcus comari CBS 809.79
Sirococcus comari CBS 807.79
Sirococcus comari CBS 806.79
Sirococcus tormentillae CBS 904.79
Sirococcus fragariae CBS 125671
Sirococcus fragariae CBS 208 34
Sirococcus fragariae CBS 121266
Sirococcus racemulus CBS 121469*
Sirococcus macounii CBS 121468
Sirococcus idaeicola CBS 125675
Sirococcus idaeicola CBS 125676
Sirococcus idaeicola CBS 125674*
Sirococcus idaeicola CBS 125672
Sirococcus idaeicola CBS 125673
Sirococcus chinensis CFCC 52286*
Sirococcus chinensis CFCC 52288
Sirococcus chinensis CFCC 52289
Sirococcus chinensis CFCC 52287
Sirococcus taishanensis SAUCC 1033
Sirococcus taishanensis CGMCC 3.28230*
Sirococcus paraclavulatus CBS 123202
Sirococcus clavulatus CBS 121255
Sirococcus smithogilvyi MUT401
Sirococcus smithogilvyi CBS 130190*
Sirococcus smithogilvyi MUT411
Sirococcus smithogilvyi CBS 130189
Sirococcus smithogilvyi CBS 130188
Sirococcus rosae CFCC 72007
Sirococcus xunwuensis CFCC 53116
Sirococcus xunwuensis CFCC 53115*
Sirococcus hainanensis CFCC 55877
Sirococcus hainanensis CFCC 54376*
Sirococcus fagacearum CFCC 54316*
Sirococcus fujianensis SAUCC 1260
Sirococcus fujianensis CGMCC 3.28229*
SAUCC 10881
Sirococcus conigenus DAOM 191767
Sirococcus conigenus CBS 113.75
Sirococcus conigenus CBS 119615*
Sirococcus conigenus CBS 119602
Sirococcus piceicola CBS 119621
Sirococcus piceicola CBS 119625
Sirococcus piceicola CBS 119620*
Sirococcus tsugae CBS 119618
Sirococcus tsugae CBS 119617
Sirococcus tsugae CBS128356
Sirococcus lanceolatae CGMCC 3.28232*
Sirococcus lanceolatae SAUCC 1277
Sirococcus euryae SAUCC 3977
Sirococcus euryae CGMCC 3.28234*
Sirococcus melastomatis SAUCC 0031
Sirococcus melastomatis CGMCC 3.28231*
Sirococcus castaneae CBS 142041
SAUCC 11015
SAUCC 11003
Sirococcus rossmaniae CFCC 54307*
Sirococcus rossmaniae CFCC 55876
Discula destructiva CBS 109771
Discula destructiva AR 2817
Apiognomonia errabunda AR 2813

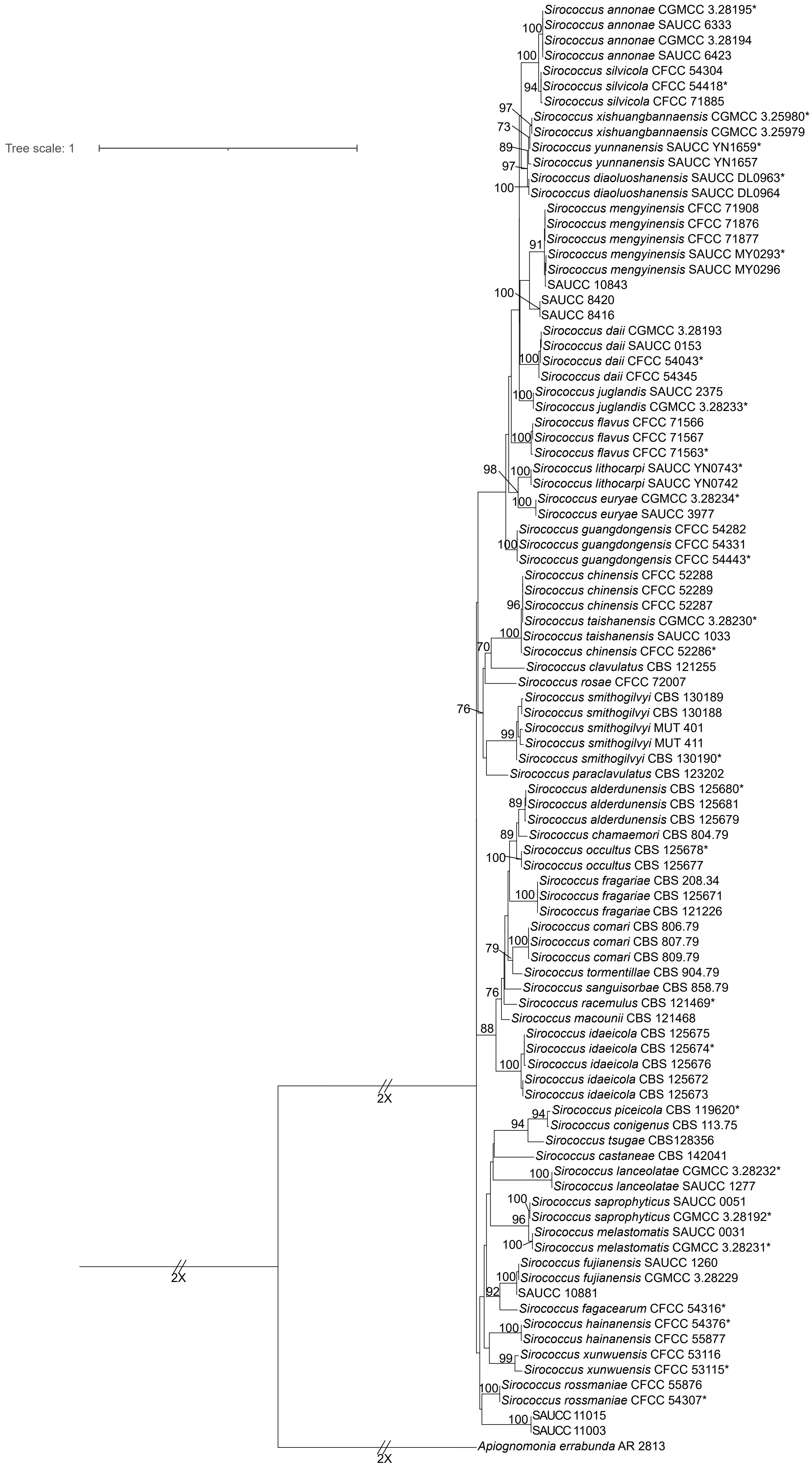

Tree scale: 1
Sirococcus annonae CGMCC 3.28195*
Sirococcus annonae SAUCC 6333
Sirococcus annonae CGMCC 3.28194
Sirococcus annonae SAUCC 6423
Sirococcus silvicola CFCC 54304
Sirococcus silvicola CFCC 54418*
Sirococcus silvicola CFCC 71885
Sirococcus xishuangbannaensis CGMCC 3.25980*
Sirococcus xishuangbannaensis CGMCC 3.25979
Sirococcus yunnanensis SAUCC YN1659*
Sirococcus yunnanensis SAUCC YN1657
Sirococcus diaoluoshanensis SAUCC DL0963*
Sirococcus diaoluoshanensis SAUCC DL0964
Sirococcus mengyinensis CFCC 71908
Sirococcus mengyinensis CFCC 71876
Sirococcus mengyinensis CFCC 71877
Sirococcus mengyinensis SAUCC MY0293*
Sirococcus mengyinensis SAUCC MY0296
SAUCC 10843
SAUCC 8420
SAUCC 8416
Sirococcus daii CGMCC 3.28193
Sirococcus daii SAUCC 0153
Sirococcus daii CFCC 54043*
Sirococcus daii CFCC 54345
Sirococcus juglandis SAUCC 2375
Sirococcus juglandis CGMCC 3.28233*
Sirococcus flavus CFCC 71566
Sirococcus flavus CFCC 71567
Sirococcus flavus CFCC 71563*
Sirococcus lithocarpi SAUCC YN0743*
Sirococcus lithocarpi SAUCC YN0742
Sirococcus euryae CGMCC 3.28234*
Sirococcus euryae SAUCC 3977
Sirococcus guangdongensis CFCC 54282
Sirococcus guangdongensis CFCC 54331
Sirococcus guangdongensis CFCC 54443*
Sirococcus chinensis CFCC 52288
Sirococcus chinensis CFCC 52289
Sirococcus chinensis CFCC 52287
Sirococcus taishanensis CGMCC 3.28230*
Sirococcus taishanensis SAUCC 1033
Sirococcus chinensis CFCC 52286*
Sirococcus clavulatus CBS 121255
Sirococcus rosae CFCC 72007
Sirococcus smithogilvyi CBS 130189
Sirococcus smithogilvyi CBS 130188
Sirococcus smithogilvyi MUT 401
Sirococcus smithogilvyi MUT 411
Sirococcus smithogilvyi CBS 130190*
Sirococcus paraclavulatus CBS 123202
Sirococcus alderdunensis CBS 125680*
Sirococcus alderdunensis CBS 125681
Sirococcus alderdunensis CBS 125679
Sirococcus chamaemori CBS 804.79
Sirococcus occultus CBS 125678*
Sirococcus occultus CBS 125677
Sirococcus fragariae CBS 208.34
Sirococcus fragariae CBS 125671
Sirococcus fragariae CBS 121226
Sirococcus comari CBS 806.79
Sirococcus comari CBS 807.79
Sirococcus comari CBS 809.79
Sirococcus tormentillae CBS 904.79
Sirococcus sanguisorbae CBS 858.79
Sirococcus racemulus CBS 121469*
Sirococcus macounii CBS 121468
Sirococcus idaeicola CBS 125675
Sirococcus idaeicola CBS 125674*
Sirococcus idaeicola CBS 125676
Sirococcus idaeicola CBS 125672
Sirococcus idaeicola CBS 125673
Sirococcus piceicola CBS 119620*
Sirococcus conigenus CBS 113.75
Sirococcus tsugae CBS128356
Sirococcus castaneae CBS 142041
Sirococcus lanceolatae CGMCC 3.28232*
Sirococcus lanceolatae SAUCC 1277
Sirococcus saprophyticus SAUCC 0051
Sirococcus saprophyticus CGMCC 3.28192*
Sirococcus melastomatis SAUCC 0031
Sirococcus melastomatis CGMCC 3.28231*
Sirococcus fujianensis SAUCC 1260
Sirococcus fujianensis CGMCC 3.28229
SAUCC 10881
Sirococcus fagacearum CFCC 54316*
Sirococcus hainanensis CFCC 54376*
Sirococcus hainanensis CFCC 55877
Sirococcus xunwuensis CFCC 53116
Sirococcus xunwuensis CFCC 53115*
Sirococcus rossmaniae CFCC 55876
Sirococcus rossmaniae CFCC 54307*
SAUCC 11015
SAUCC 11003
Apiognomonia errabunda AR 2813
2X
2X
2X
